# Comparative risk of tuberculosis infection with different TNF-α inhibitors in immune-mediated inflammatory diseases: a systematic review and network meta-analysis

**DOI:** 10.3389/fimmu.2026.1726299

**Published:** 2026-01-29

**Authors:** Xiuying Lv, Yuan Liu, Yan Li, Qi Zhang, Shiju Chen, Xiaomei Liu, Guixiu Shi, Yan Li

**Affiliations:** 1 Department of Rheumatology and Clinical Immunology, the First Affiliated Hospital of Xiamen University, School of Medicine, Xiamen University, Xiamen, China; 2 Xiamen Municipal Clinical Research Center for Immune Diseases, Xiamen, China; 3 Xiamen Key Laboratory of Rheumatology and Clinical Immunology, Xiamen, China; 4 Department of Rheumatology and Immunology, The First Affiliated Hospital of Zhejiang Chinese Medical University, Hangzhou, China

**Keywords:** immune-mediated inflammatory diseases, network meta-analysis, systematic review, TNF-α inhibitors, tuberculosis

## Abstract

**Background:**

Tumor necrosis factor-α inhibitors (TNFi) are established to increase the risk of tuberculosis (TB). However, the comparative risk across different TNFi agents remains poorly defined due to a lack of head-to-head comparative studies. This network meta-analysis (NMA) aimed to evaluate and compare the risk of TB infection associated with various TNFi therapies in patients with immune-mediated inflammatory diseases (IMIDs) based on real-world, long-term cohort studies.

**Methods:**

We conducted a systematic search of PubMed, EMBASE, Cochrane Library, and Web of Science from inception to May 30, 2025, for cohort studies reporting TB events in patients with IMIDs treated with TNFi. Study selection, data extraction, and risk of bias assessment were performed by three independent reviewers using the Newcastle-Ottawa Scale. A Bayesian arm-based NMA with random-effects models was used to estimate log risk ratio (logRR) and 95% credible intervals (CrIs) for TB infection across different TNFi agents compared with TNFi-naive.

**Results:**

A total of 19 cohort studies involving 396, 044 patients were included. Compared to TNFi-naive, infliximab (IFX) was associated with the highest risk of TB (logRR = 2.32, 95% CrI: 1.12-3.32), followed by adalimumab (ADA) (logRR = 1.72, 95% CI: 0.42-2.65) and etanercept (ETN) (logRR = 1.39, 95% CI: 0.33-2.42). Certolizumab pegol (CZP) was associated with the lowest risk among TNFi agents.

**Conclusion:**

TNFi treatment in patients with IMIDs is associated with a significantly increased risk of TB infection. Among the TNFi agents, IFX was associated with the highest risk, while ETN and CZP demonstrated lower risks. These findings can inform clinical decision-making, suggesting that ETN or CZP may be preferable in patients with high TB risk, while emphasizing that vigilant TB monitoring remains paramount regardless of the chosen agent.

**Systematic Review Registration:**

https://www.crd.york.ac.uk/prospero/, identifier CRD42022331674.

## 1 Introduction

Immune-mediated inflammatory diseases (IMIDs) encompass a heterogeneous group of prevalent conditions, including rheumatoid arthritis (RA), spondyloarthropathy (SpA), connective tissue disorders, inflammatory cutaneous conditions, and inflammatory bowel disease (IBD), etc (1). Inadequate disease control can lead to progressive disability, loss of work capacity, reduced quality of life, and substantial socioeconomic burdens (2). While the precise pathogenesis of IMIDs is not fully elucidated, TNF-α existing in both soluble (sTNF-α) and transmembrane (tmTNF-α) forms has been identified as a key pro-inflammatory cytokine driving disease pathology (3). Consequently, TNFi which block the inflammatory effects of TNF-α, are widely used in the treatment of IMIDs and have significantly improved clinical outcomes (4). Currently, five TNFi agents are approved for clinical use: etanercept (ETN), adalimumab (ADA), infliximab (IFX), golimumab (GOL), and certolizumab pegol (CZP). IFX, ADA, and GOL are full-length IgG1 monoclonal antibodies against TNF-α. ETN is a fusion protein consisting of the extracellular domain of the human TNF receptor 2 (TNFR2) linked to the Fc portion of human IgG1. CZP is a PEGylated Fab′ fragment of a humanized anti-TNF-α monoclonal antibody and lacks the Fc region (5). The widespread use of TNFi has been accompanied by growing concerns regarding associated infections, particularly tuberculosis (TB) (6). However, due to the absence of head-to-head comparative trials, direct evidence comparing the TB risk among different TNFi agents is limited. Previous systematic reviews and meta-analyses have reported that the risk of TB in patients treated with TNFi is 1.6 to 25.1 times higher than that in the general population (7–12). Subgroup analyses suggest that monoclonal antibody-based TNFi agents confer a higher risk of TB than soluble receptor analogs (7–9). Some randomized controlled trials (RCTs) have shown no significant difference in TB incidence between IFX and ETN in RA patients (13, 14), however, these studies were limited by small sample sizes and short follow-up durations, which may not accurately reflect real-world risk differences. Existing systematic reviews and meta-analyses are predominantly based on RCTs, which often involve homogeneous patient populations, fixed drug regimens, and relatively short treatment durations. In contrast, rheumatic diseases are chronic and complex, frequently requiring long-term treatment and individualized therapy adjustments based on disease activity factors that complicate the accurate assessment of TB risk. Furthermore, most available data pertain to RA, with limited information on other IMIDs.

To better reflect clinical practice, this meta-analysis incorporated cohort studies involving patients with various IMIDs treated with TNFi and followed for at least one year, thereby providing a more robust evaluation of TB risk. This network meta-analysis (NMA) therefore aims to synthesize long-term, real-world evidence from diverse global populations to provide a comprehensive ranking of TB risk among all five TNFi agents, ultimately offering higher-quality evidence to guide individualized clinical decision-making, particularly in TB-endemic areas.

Registration: This systematic review and meta-analysis was registered on PROSPERO (CRD42022331674, Last updated:2025.12.25).

## 2 Materials and methods

### 2.1 Protocol

This systematic review and NMA were conducted in accordance with the Preferred Reporting Items for Systematic Reviews and Meta-Analyses (PRISMA) guidelines (http://www.prisma-statement.org/) and registered in the PROSPERO database (CRD42022331674, Last updated:2025.12.25). The PRISMA 2020 Checklist was provided in **Supplementary file 2**.

### 2.2 Search strategy

A systematic literature search was conducted independently by two investigators (Q.Z. and Y.L.¹) in PubMed, EMBASE, the Cochrane Library, and Web of Science for English-language cohort studies published from inception to May 30, 2025. The search strategy combined Medical Subject Headings (MeSH) and keywords related to TNFi agents including etanercept, adalimumab, infliximab, golimumab, certolizumab pegol, TNF-α antagonist. The detailed search strategy is provided in **Supplementary Table S1**.

### 2.3 Eligibility criteria

Studies were included if they met the following criteria: (1) cohort studies including at least two cohorts of patients with IMIDs aged ≥18 years treated with different TNFi agents; (2) reported TB incidence; and (3) had a follow-up period of ≥1 year. When multiple publications from the same study population existed, only the most comprehensive or extended report was included. Studies with overlapping data were excluded.

### 2.4 Study selection and data extraction

Two reviewers (X.L. and Y.L.^123^) independently screened titles and abstracts, reviewed full texts, and extracted data using a standardized form. The extracted information included first author, publication year, country, study period, sample size, percentage of female participants, and TB incidence. Any discrepancies were resolved through discussion or by consulting a third reviewer (S.C.).

### 2.5 Risk of bias assessment and quality of evidence

Publication bias was evaluated using funnel plots and Egger’s test (15). Two investigators (Y.L.¹ and X.L.) independently assessed the methodological quality of the included studies using the Newcastle-Ottawa Scale (NOS) for cohort studies. The NOS evaluated three domains: selection of study groups, comparability of groups, and outcome assessment. Studies were awarded a maximum of 9 stars, with higher scores indicating higher quality. Disagreements were resolved by consensus or by involving a third reviewer (S.C.).

### 2.6 Statistical analysis

We performed a Bayesian arm-based NMA using the “BUGSnet” package (16) in R (version 4.1.0; R Foundation for Statistical Computing). Markov chain Monte Carlo (MCMC) sampling was implemented using JAGS (17). Both fixed-effect and random-effects models were fitted, and model fit was compared using the deviance information criterion (DIC). Models with lower DIC values were preferred. Heterogeneity was assessed using the I² statistic and chi-square tests, where I² > 50% or p < 0.10 indicated substantial heterogeneity (18). Sensitivity analyses determined the effect of individual studies by sequential exclusion. Treatment rankings were estimated using the surface under the cumulative ranking curve (SUCRA). LogRR and 95% CrIs for TB infection across different TNFi agents compared with TNFi-naive were estimated. Subgroup analyses were conducted based on follow-up duration and the use of prophylactic anti-TB therapy. Consistency between direct and indirect evidence was assessed by comparing consistency and inconsistency models.

## 3 Results

### 3.1 Search results and study characteristics

**Figure 1:**
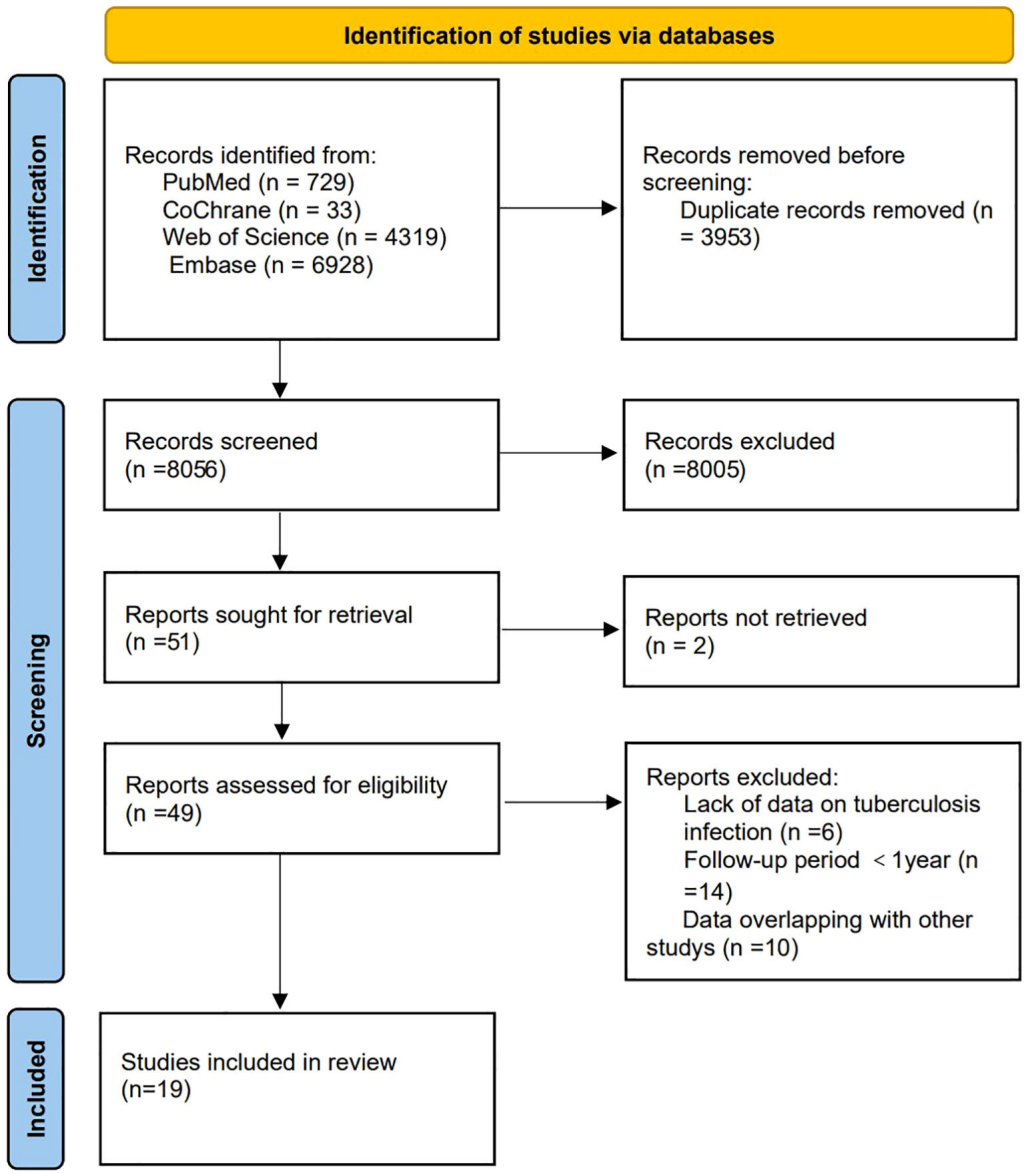
Flow diagram of study identification, screening, eligibility assessment, and inclusion. This study ultimately included 19 cohort studies that met the criteria.

The initial search identified 12, 009 articles. After screening, 19 cohort studies (19–37) involving 396, 044 patients were included in the analysis (**Figure 1**). The studies were published between 2004 and 2024 and conducted in 15 countries. The characteristics of the included studies were summarized in **Table 1**. All included studies had NOS scores ≥ 6, indicating moderate to high quality (**Table 2**). For the study by Lee et al. (2021) (34), data were extracted specifically for the patient cohort receiving only one TNF inhibitor to maintain consistency in the exposure definition across studies. Patients who received multiple biologics were excluded from our quantitative synthesis.

**Table 1:** The characteristics of the cohort studies included in the meta-analysis.

Study	Country	Disease	Treatment	Number of patients	Month of follow-up	Year	Female, %	Preventive anti-TB
Wallis RS, 2004 (19)	United States	Unrestricted	ETN	113000	36	58 (52–68)	59	No
			IFX	233000	36	60 (46–68)	66	
Listing J, 2005 (20)	Germany	RA	TNF-naive	601	12	56.5 ± 11.4	82.7	No
			ETN	512	12	53.7 ± 12.6	78.1	
			IFX	346	12	53.6 ± 12.6	70.8	
Sichletidis L, 2006 (21)	Greece	RA, AS, IBD, PSA	ETN	202	35	49 ± 8	67.7	Yes
			IFX	298	35	49 ± 8	67.7	
			ADA	113	35	49 ± 8	67.7	
Fernandez-Nebro A, 2007 (22)	Spain	RA	ETN	79	24	54.0 ± 12.4	81	No
			IFX	60	24	54.0 ± 11.6	81	
			ADA	22	24	54.0 ± 10.4	81	
Favalli EG, 2009 (23)	Italy	RA	ETN	242	23.22	55.81 ± 14.57	84.3	No
			IFX	519	29.88	55.72 ± 12.07	81.5	
			ADA	303	21.26	56.07 ± 13.11	85.1	
Fidder H, 2009 (24)	Belgium	IBD	TNF -naive	666	144	46 (37–55)	50	No
			IFX	743	58	40 (31–50)	57	
Dewedar AM, 2012 (25)	Saudi Arabia	RA	TNF -naive	112	60	46.09 ± 12.55	87.5	No
			ETN	20	60	35.05 ± 12.14	87.5	
			IFX	56	60	35.05 ± 12.14	87.5	
			ADA	36	60	35.05 ± 12.14	87.5	
Lee SK, 2013 (26)	Korean	RA	ETN	170	13	43 (18–85)	52.7	No
			IFX	119	13	43 (18–85)	52.7	
			ADA	67	13	43 (18–85)	52.7	
Yoo IK, 2014 (27)	Korean	RA, AS, PsA, IBD	IFX	72	22	40.9	34.8	Yes
			ADA	103	22	40.9	34.8	
Kim M, 2016 (28)	Korean	AS	ETN	32	86.0 ± 36.0	44.0 ± 14.0	18.8	Yes
			IFX	66	53.6 ± 23.3	43.0 ± 12.0	33.3	
			ADA	45	84.8 ± 46.2	36.2 ± 12.8	40	
Lim CH, 2016 (29)	China, TaiWan	RA	TNF -naive	32094	12	55 ± 13	80.3	No
			ETN	2925	12	55 ± 13	80.3	
			ADA	2424	12	55 ± 13	80.3	
Cagatay T, 2017 (30)	Turkey	Unrestricted	ETN	659	28.1 ± 28.1	TB: 38.5 ± 9.2 Non-TB: 42.41 ± 12.71	51.9	Yes
			IFX	864	28.1 ± 28.1		51.9	
			ADA	364	28.1 ± 28.1		51.9	
Shobha V, 2018 (31)	India	Unrestricted	ETN	61	12	41 (16‐82)	50.26	Yes
			IFX	41	12	41 (16‐82)	50.26	
			ADA	25	12	41 (16‐82)	50.26	
Sousa M, 2019 (32)	Portugal	IBD	IFX	92	21.6	60	56	No
			ADA	24	21.6	60	56	
			GOL	1	21.6	60	56	
Argüder E, 2020 (33)	Turkey	Unrestricted	ETN	156	36	47.45 ± 11.85	61.3	Yes
			IFX	30	36	47.45 ± 11.85	61.3	
			ADA	108	36	47.45 ± 11.85	61.3	
			GOL	50	36	47.45 ± 11.85	61.3	
			CZP	49	36	47.45 ± 11.85	61.3	
Lee JY, 2021 (34)	Korea	IBD	IFX	939	48.54 ± 36.48	31.35 ± 13.59	34.4	Yes
			ADA	311	48.54 ± 36.48	31.35 ± 13.59	34.4	
			GOL	9	48.54 ± 36.48	31.35 ± 13.59	34.4	
Koo BS, 2021 (35)	France	AS	ETN	528	17.88	NA	19.56	No
			IFX	445	21.12	NA	18.43	
			ADA	914	18.24	NA	17.94	
			GOL	628	17.52	NA	18.95	
Slouma M, 2022 (36)	Tunisia	RA, SpA	ETN	12	16.7 ± 0.9	42 ± 3.4	54.9	Yes
			IFX	20	16.7 ± 0.9	42 ± 3.4	54.9	
			ADA	11	16.7 ± 0.9	42 ± 3.4	54.9	
			GOL	3	16.7 ± 0.9	42 ± 3.4	54.9	
Boqaeid A, 2024 (37)	Saudi	Unrestricted	ETN	262	21.5 ± 8.4	47.92 ± 17.1	79.8	No
			ADA	391	36 ± 8.9	38.4 ± 17.7	58.8	

The characteristics of the cohort studies included in the meta-analysis. RA, rheumatoid arthritis; AS, Ankylosing Spondylitis; IBD, inflammatory bowel disease; PsA, psoriatic arthritis; SpA, spondyloarthropathy; ETN, Etanercept; ADA, Adalimumab; IFX, infliximab; GOL, golimumab; CZP, certolizumab pegol.

**Table 2:** The Newcastle-Ottawa scale served to assess the risk of bias in the included studies.

Study	Selection				Comparability	Outcome			Score
	Representativeness of the exposed cohort	Selection of the non-exposed cohort	Ascertainment of exposure	Demonstration that outcome of interest was not present at start of study	Comparability of cohorts on the basis of the design or analysis controlled for confounders	Assessment of outcome	Was follow-up long enough for outcomes to occur	Adequacy of follow-up of cohorts	
Wallis RS, 2004 (19)	★	★	★	★		★	★	★	7
Listing J, 2005 (20)	★	★	★	★		★	★		6
Sichletidis L, 2006 (21)	★	★	★	★	★	★	★		7
Fernandez-Nebro A, 2007 (22)	★	★	★	★	★	★	★	★	8
Favalli EG, 2009 (23)	★	★	★		★	★	★	★	7
Fidder H, 2009 (24)	★	★	★	★	★	★	★	★	8
Dewedar AM, 2012 (25)	★	★	★	★	★★	★	★	★	9
Lee SK, 2013 (26)	★	★	★		★	★	★	★	7
Yoo IK, 2014 (27)	★	★	★		★	★	★	★	7
Kim M, 2016 (28)	★	★	★		★	★		★	6
Lim CH, 2016 (29)	★	★	★	★	★	★	★	★	8
Cagatay T, 2017 (30)	★	★	★		★	★	★	★	7
Shobha V, 2018 (31)	★		★	★	★	★	★		6
Sousa M, 2019 (32)	★		★	★	★	★	★	★	7
Argüder E, 2020 (33)	★		★	★	★	★	★	★	7
Lee JY, 2021 (34)	★	★	★	★	★	★	★	★	8
Koo BS, 2021 (35)	★		★	★	★	★	★	★	7
Slouma M, 2022 (36)		★	★	★	★★	★	★	★	8
Boqaeid A, 2024 (37)	★		★	★	★★	★	★	★	8

### 3.2 Network geometry

**Figure 2:**
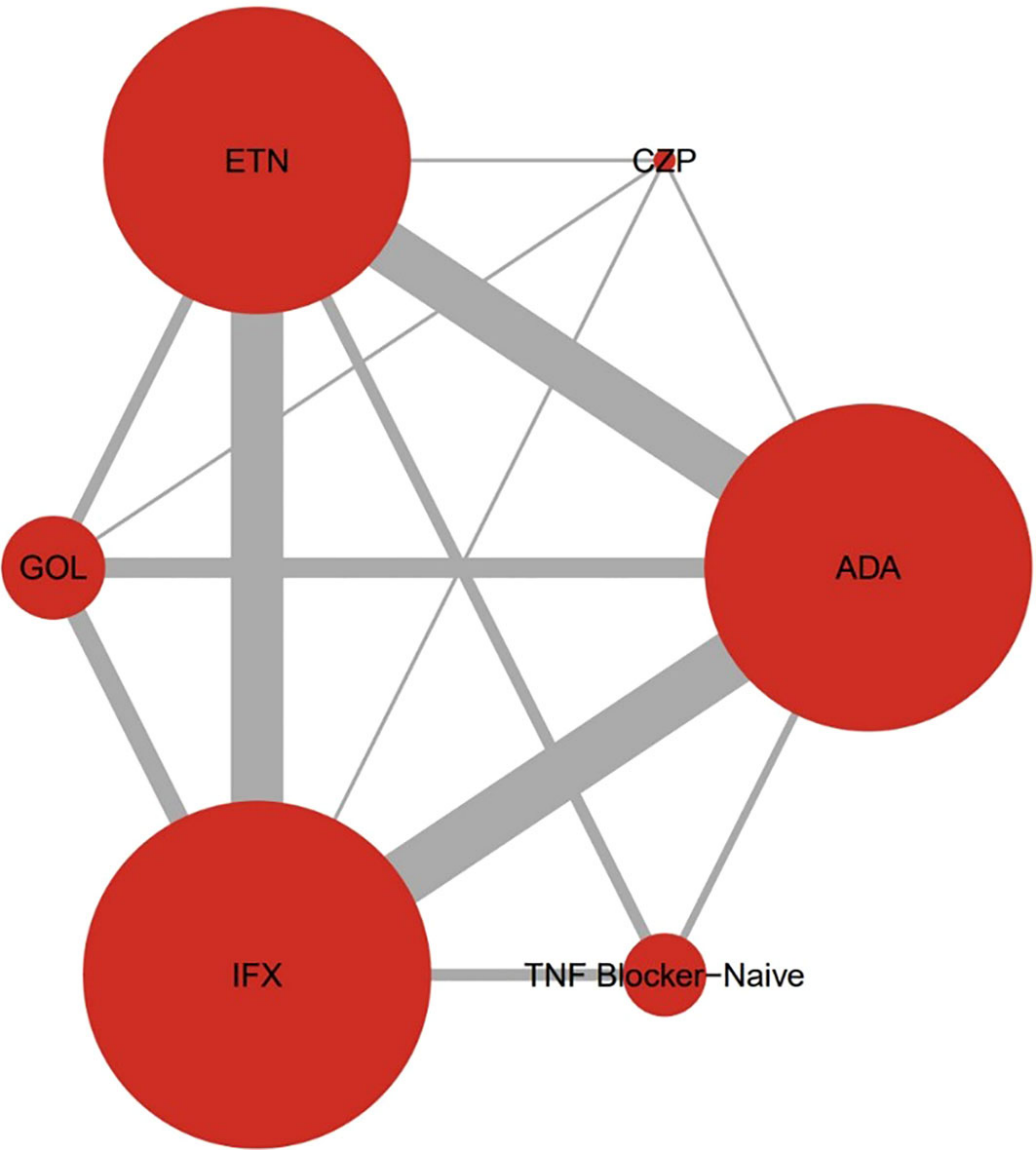
The network diagram of this meta-analysis. Closed loops were detected between different treatment groups. Closed loops referred to direct comparisons including more than 2 comparators. The size of the nodes was proportional to the number of comparisons involving that treatment node, while the thickness of the edges indicated the number of studies that included the 2 connected treatments. ETN, Etanercept; ADA, Adalimumab; IFX, Infliximab; GOL, Golimumab; CZP, Certolizumab pegol.

The network plot of treatment comparisons was shown in **Figure 2**. The size of each node corresponds to the number of patients receiving that treatment, and the thickness of the edges represents the number of studies comparing connected treatments. Closed loops indicated the presence of direct comparisons involving more than two treatments. The network included multiple closed loops, with the most frequent direct comparison being between IFX and ADA.

### 3.3 Model selection and consistency

**Figure 3:**
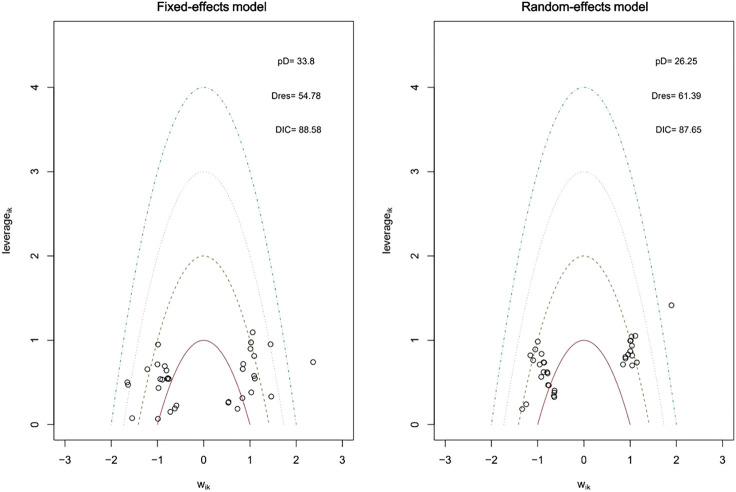
Leverage plots and DIC for fixed and random effects models for TB infection. Regarding outliers, data points falling outside the purple arc suggested they may lead to poor model fit. A lower DIC value indicated a better model fit. DIC, deviance information criterion; Dres, deviance residual; pD, posterior mean deviance; Wik, adjustment for normal distribution of studies and arms; TB, Tuberculosis.

**Figure 4:**
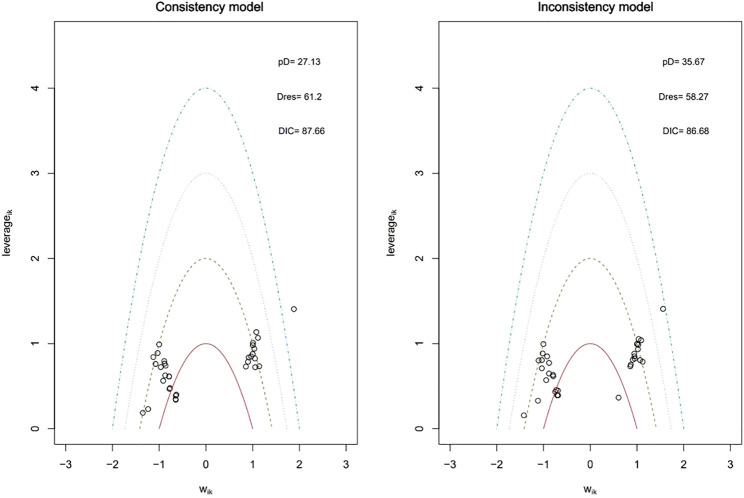
Leverage plots and DIC for consistency and inconsistency model for TB. The inconsistency model had slightly less DIC than the consistency model. Regarding outliers, data points falling outside the purple arc suggested they may lead to poor model fit. A lower DIC value indicated a better model fit. DIC, deviance information criterion; Dres, deviance residual; pD, posterior mean deviance; Wik, adjustment for normal distribution of studies and arms; TB, Tuberculosis.

The random-effects model demonstrated a better fit (lower DIC) than the fixed-effect model (**Figure 3**) and was therefore selected for the primary analysis. Heterogeneity across the studies was significant (I² > 50%, p < 0.1, see **Supplementary Figures S2**), which was anticipated given the inclusion of diverse IMIDs and varying background TB risk across geographical regions. In terms of risk of bias, most studies were rated with some concerns (see **Supplementary Figures S1, S2**). The random-effects model was chosen to account for this clinical and methodological heterogeneity. Consistency between direct and indirect evidence was assessed using inconsistency models, we found that the inconsistency models had slightly less DIC than the consistency mode (**Figure 4**). This illustrates the possibility of inconsistency in the network.

### 3.4 Treatment rankings and league table

**Figure 5:**
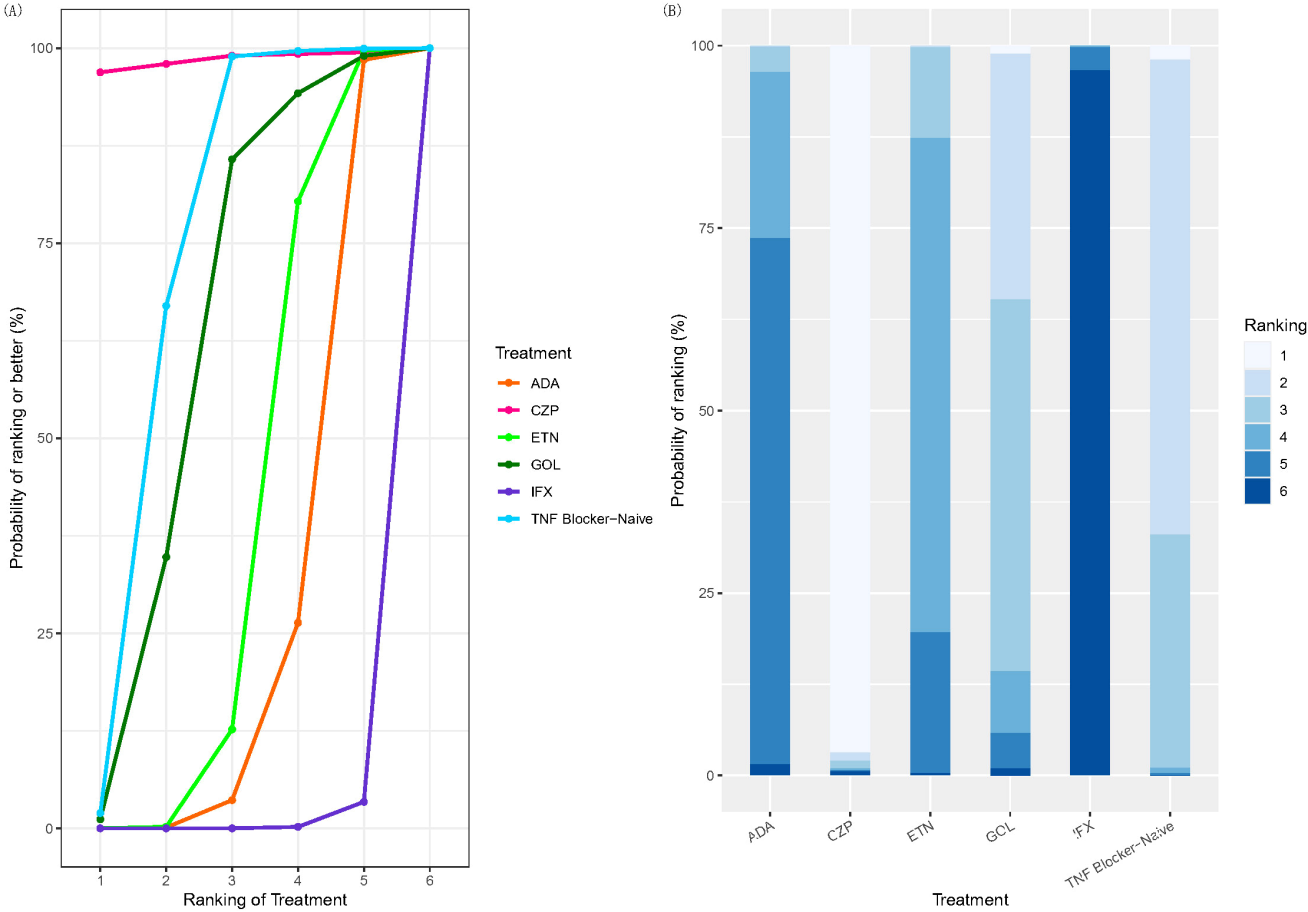
SUCRA plot and plot of treatment rank probabilities. **(A)** TB SUCRA plot. Higher rankings associated with smaller outcome values. **(B)** plot of treatment rank probabilities. Treatments: ETN, Etanercept; ADA, Adalimumab; IFX, Infliximab; GOL, Golimumab; CZP, Certolizumab pegol; TB, Tuberculosis; SUCRA, surface under the cumulative ranking curve.

**Figure 6:**
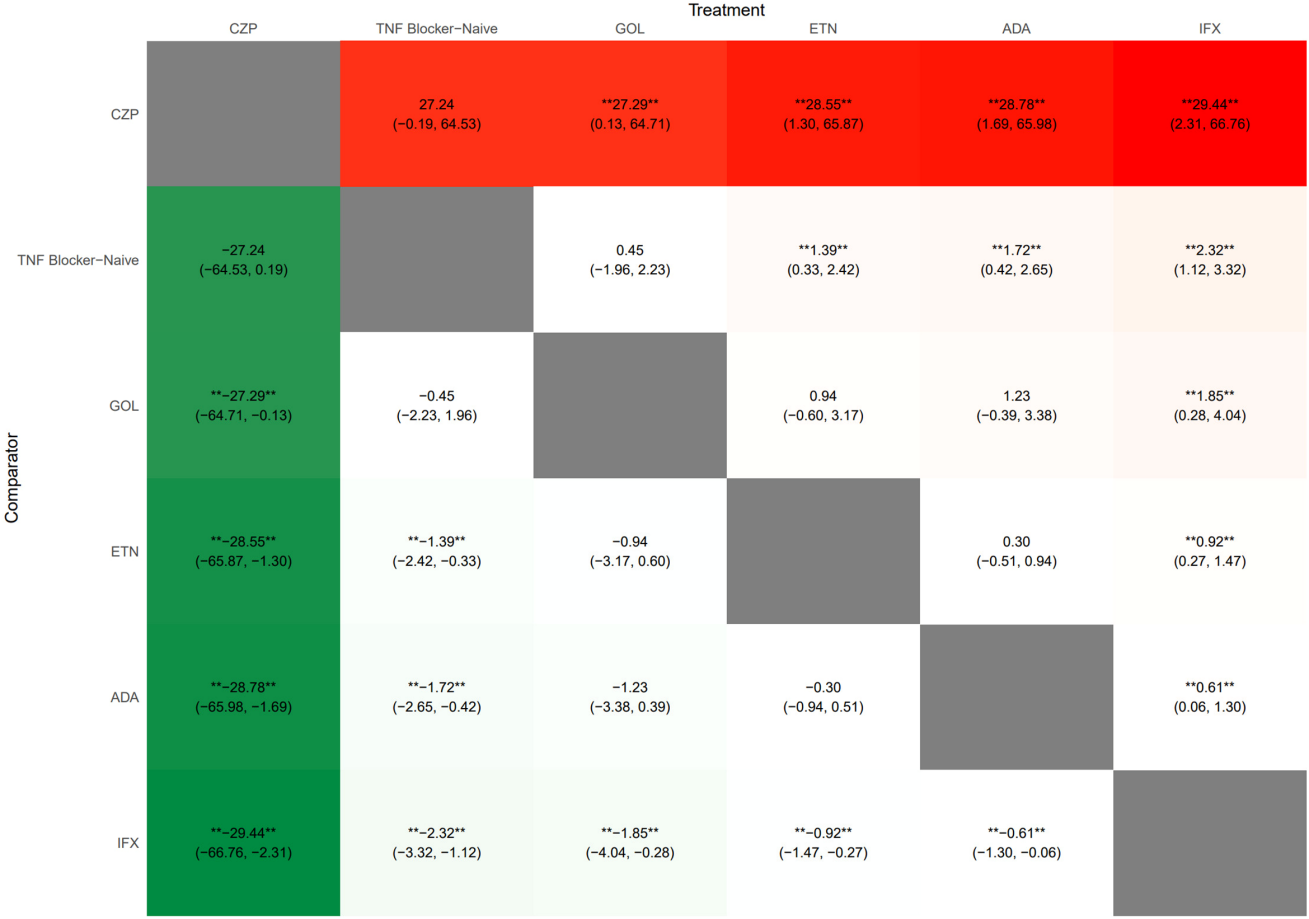
League heat plot for all treatment in the network for TB. The league plot provided a comprehensive summary of the NMA results, indicating the significance of all interventions compared to both the TNF blocker-naive and other treatments. Each cell showed the outcome for the row intervention relative to the corresponding column intervention. Green cells indicated that the row intervention carried a lower risk of tuberculosis infection than the column intervention, whereas red cells signified a higher associated risk. The symbols (**) denoted statistically significant differences between treatments and comparators at a 95% confidence level. The negative values represented beneficial or protective associations, while positive values represented adverse or harmful associations. ETN, Etanercept; ADA, Adalimumab; IFX, Infliximab; GOL, Golimumab; CZP, Certolizumab pegol; TB, Tuberculosis.

SUCRA values and rank probabilities consistently identified IFX as the TNFi agent associated with the highest risk of TB, followed by ADA and then ETN. CZP demonstrated the most favorable (lowest risk) profile among the TNFi agents evaluated (**Figure 5**). The league table (**Figure 6**) presented pairwise comparisons between treatments. Each cell showed the outcome for the row intervention relative to the corresponding column intervention. In this plot, green cells signified that the row intervention was associated with a lower risk of TB infection compared with the column intervention, whereas red cells signified a higher associated risk. The symbols (**) denoted statistically significant differences between treatments and comparators at a 95% confidence level.

**Figure 7:**
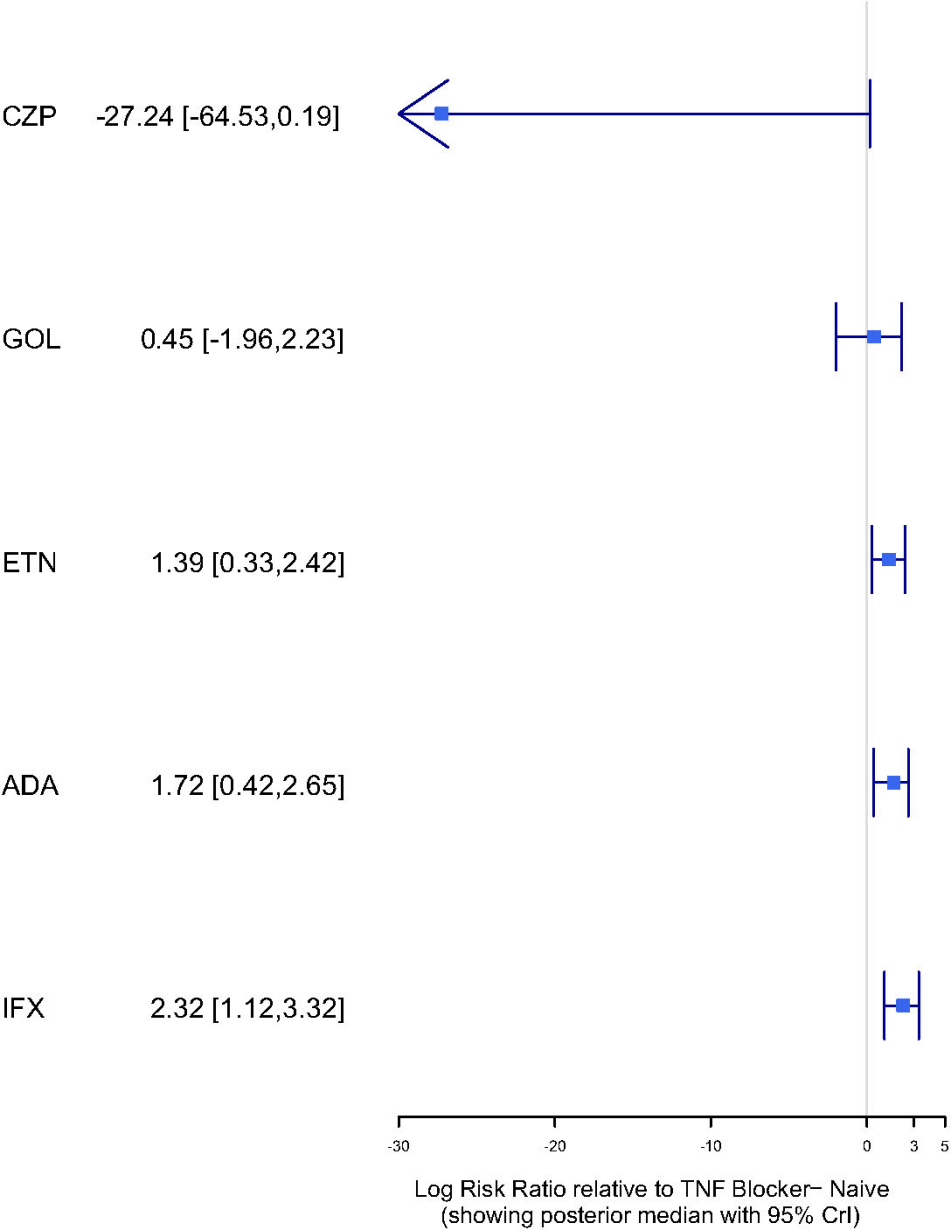
The forest plot of the logRR in different treatment compared to TNF blocker-naive. The result showed the LogRR of patients with IMIDs receiving different TNFi versus TNF blocker-naive. The negative values represented beneficial or protective associations, while positive values represented adverse or harmful associations. ETN, Etanercept; ADA, Adalimumab; IFX, Infliximab; GOL, Golimumab; CZP, Certolizumab pegol.

There were with significant differences observed between IFX and CZP (logRR = 29.44, 95% CI: 2.31–66.76) and between ADA and CZP (logRR = 28.78, 95% CI: 1.69–65.98). Compared with TNFi-naive patients, IFX (logRR = 2.32, 95% CI: 1.12–3.32), ADA (logRR = 1.72, 95% CI: 0.42–2.65), and ETN (logRR = 1.39, 95% CI: 0.33–2.42) were associated with significantly increased risks of TB. No significant differences were observed for GOL or CZP compared with TNFi-naive patients. Forest plots illustrating these comparisons are shown in **Figure 7**.

### 3.5 Subgroup analyses and sensitivity analysis

Subgroup analyses based on follow-up duration (<2 years) and prophylactic anti-TB therapy yielded results consistent with the main analysis (see **Supplementary Figures S3 and S4**). The sensitivity analysis in the meta-analysis indicated that the exclusion of individual studies had little impact on the results. This suggests that the findings of the meta-analysis are robust and not significantly influenced by any single study (see **Supplementary Figures S5**).

## 4 Discussion

This systematic review and NMA provided a comprehensive comparative safety assessment of five TNFi agents regarding the risk of TB infection in patients with IMIDs based on real-world, long-term cohort data.

The principal finding that IFX carries the highest risk of TB, followed by ADA and then ETN, with CZP appearing to have the lowest risk, is consistent with the prevailing hypothesis that monoclonal antibodies confer a greater risk than soluble receptor constructs. This risk hierarchy was robust, remaining consistent across subgroup analyses of follow-up duration and prophylactic anti-TB therapy.

### 4.1 Mechanistic insights into differential TB risk

The observed differential risk profile can be plausibly explained by distinct mechanisms of action among TNFi agents. TNF-α is a critical cytokine for maintaining the structural integrity of granulomas, which are essential for containing Mycobacterium tuberculosis infection (38–40). Beyond sTNF-α, monoclonal antibodies such as IFX, ADA, GOL also bind tmTNF-α with high affinity. This binding can induce complement-dependent cytotoxicity (CDC) and antibody-dependent cellular cytotoxicity (ADCC), leading to the lysis of immune cells such as, monocytes and T-cells which express tmTNF-α and are crucial for granuloma stability (41–43). This lytic effect potentially disrupts existing granulomas, facilitating bacterial dissemination and reactivation of latent TB.

In contrast, ETN, a soluble receptor fusion protein, has a lower binding avidity for tmTNF-α and lacks an Fc domain capable of effectively recruiting complement or effector cells, resulting in markedly reduced CDC/ADCC activity (43, 44). CZP, a PEGylated Fab’ fragment, completely lacks an Fc region, which explains its absence of CDC activity and may account for its seemingly favorable risk profile in our analysis (45). Furthermore, evidence suggests that monoclonal antibodies, by compromising the key function of signaling through tmTNF-α, impair the innate immune control of TB. This function is preserved by receptor agonists. This mechanism provides a distinct explanation for the increased TB risk associated with monoclonal antibodies (41, 46). In recent years, researchers have also explored the association between HLA-B subtypes and tuberculosis development induced by anti-TNF therapy from the perspective of genetic susceptibility (47).

### 4.2 Addressing heterogeneity and limitations

A primary strength of our study is its inclusion of diverse IMIDs and global populations, which enhances the generalizability of our findings. However, this diversity inevitably introduces clinical heterogeneity. Variations in background TB incidence rates ranging from low to high burden across the included countries, differences in standard-of-care practices such as screening protocols and prophylactic therapy, and the spectrum of concomitant immunosuppressants including corticosteroids and csDMARDs could all contribute to the observed statistical heterogeneity (I² > 50%). We addressed this by employing a Bayesian random-effects model, which is explicitly designed to account for such variability, providing more conservative and generalizable effect estimates. Nevertheless, this heterogeneity necessitates cautious interpretation of the point estimates.

Several other limitations warrant consideration. First, the evidence base for CZP and GOL is notably scarce. Particularly for CZP, the estimate relied on only one small study comprising 49 patients. The point estimate for CZP suggests a potentially lower risk, but the exceedingly wide credible intervals indicate very low certainty in this estimate. These findings are therefore primarily hypothesis-generating, and more robust data from large-scale prospective studies are needed to draw definitive conclusions regarding the TB risk associated with CZP and GOL. Second, while we focused on comparative safety, treatment efficacy was not evaluated. A comprehensive clinical decision must balance the TB risk identified here against the known differential efficacy of these drugs for specific IMIDs. Future studies integrating both efficacy and safety outcomes are valuable. Finally, as with any meta-analysis, our results are constrained by the quality and reporting of the original studies.

### 4.3 Clinical and research implications

Despite these limitations, our findings have tangible clinical implications. In patients with significant risk factors for TB reactivation (e.g., from high-burden regions, prior latent TB infection, or on concomitant steroids) (48), opting for a soluble receptor inhibitor or CZP may be a prudent choice when clinically appropriate, potentially mitigating the risk of this serious infection. This decision must be made within the context of individual patient factors, disease severity, and drug availability. Our study also underscores the non-interchangeable nature of TNFi agents from a safety perspective.

From a research perspective, our work highlights the critical need for large, prospective pharmacovigilance studies that directly compare newer agents like GOL and CZP against established ones. Furthermore, translational research exploring the precise immunologic mechanisms, especially those involving the role of Fc-mediated functions and tmTNF-α signaling in granuloma biology, will be crucial for understanding the differential risks observed in epidemiological studies and for guiding the development of safer biologic therapies.

## 5 Conclusion

In conclusion, this NMA demonstrates a gradient of TB risk among TNFi agents used for IMIDs. The risk is highest with the monoclonal antibody IFX, intermediate with ADA, and lower with the soluble receptor fusion protein ETN. This spectrum aligns with understood differences in their mechanisms of action, particularly their capacity to induce cytolytic effects on tmTNF-α-expressing cells. While heterogeneity exists and evidence for some agents remains limited, these findings provide valuable guidance for clinicians in stratifying TB risk and making individualized treatment decisions, especially in TB-endemic areas. Ultimately, vigilant screening for latent TB and maintaining a high index of suspicion for active infection remain paramount, regardless of the chosen TNFi agent.

## Data Availability

The original contributions presented in the study are included in the article/**Supplementary Material**. Further inquiries can be directed to the corresponding authors.
