# Supplementary material for: Comparative risk of tuberculosis infection with different TNF-α inhibitors in immune-mediated inflammatory diseases: a systematic review and network meta-analysis
Source: Front Immunol. 2026 Jan 29;17:1726299. doi: 10.3389/fimmu.2026.1726299 (PMC12894354; doi:10.3389/fimmu.2026.1726299)
Supplement: Supplementary file 1 [file Supplementaryfile1.docx]

**Supplementary Materials**

Table S1. The Search Strategy of each database.

Figure S1. Funnel plots of the effects of intervention arms on TB

Figure S2. Forest plots from pairwise meta-analyses of the relative risk (RR) for tuberculosis (TB) treatment outcomes.

Figure S3. Surface under the cumulative ranking curve (SUCRA) and treatment rank probabilities for tuberculosis (TB) among patients with a follow-up duration of less than 2 years.

Figure S4. Surface under the cumulative ranking curve (SUCRA) and treatment rank probabilities for tuberculosis (TB) among patients with prophylactic anti-TB therapy.

Figure S5. Sensitivity analysis of the meta-analysis results for tuberculosis (TB) treatment: sequential omission of individual intervention pairs.

Supplementary Table1. The Search Strategy of each database.

| Database | Search number | Search Details | Results |
| --- | --- | --- | --- |
| **Pubmed** | 23 | ((((("Tumor Necrosis Factor Inhibitors"[MeSH Terms] AND 1000/01/01:2025/05/30[Date - Publication]) OR (("tumor necrosis factor blockers"[Title/Abstract] OR "tnf inhibitors"[Title/Abstract] OR "inhibitors tnf"[Title/Abstract] OR "tnf blockers"[Title/Abstract] OR "blockers tnf"[Title/Abstract] OR "tumor necrosis factor blocker"[Title/Abstract] OR "tumor necrosis factor antagonist"[Title/Abstract] OR "tumor necrosis factor inhibitor"[Title/Abstract] OR "tnf antagonist"[Title/Abstract] OR "antagonist tnf"[Title/Abstract] OR "tnf blocker"[Title/Abstract] OR (("Blocker"[All Fields] OR "blocker s"[All Fields] OR "Blockers"[All Fields]) AND "TNF"[Title/Abstract]) OR "tnf inhibitor"[Title/Abstract] OR "inhibitor tnf"[Title/Abstract] OR "tumor necrosis factor antagonists"[Title/Abstract] OR "tnf antagonists"[Title/Abstract] OR "antagonists tnf"[Title/Abstract] OR (((("cysts"[MeSH Terms] OR "cysts"[All Fields] OR "cyst"[All Fields] OR "neurofibroma"[MeSH Terms] OR "neurofibroma"[All Fields] OR "neurofibromas"[All Fields] OR "tumor s"[All Fields] OR "tumoral"[All Fields] OR "tumorous"[All Fields] OR "tumour"[All Fields] OR "neoplasms"[MeSH Terms] OR "neoplasms"[All Fields] OR "Tumor"[All Fields] OR "tumour s"[All Fields] OR "tumoural"[All Fields] OR "tumourous"[All Fields] OR "tumours"[All Fields] OR "tumors"[All Fields]) AND ("necrose"[All Fields] OR "necrosed"[All Fields] OR "necrosi"[All Fields] OR "necrosing"[All Fields] OR "Necrosis"[MeSH Terms] OR "Necrosis"[All Fields] OR "necroses"[All Fields]) AND ("factor a"[Supplementary Concept] OR "factor a"[All Fields])) AND ("tnf protein human"[Supplementary Concept] OR "tnf protein human"[All Fields] OR "tnf a"[All Fields])) AND "Antagonists"[Title/Abstract]) OR (((("cysts"[MeSH Terms] OR "cysts"[All Fields] OR "cyst"[All Fields] OR "neurofibroma"[MeSH Terms] OR "neurofibroma"[All Fields] OR "neurofibromas"[All Fields] OR "tumor s"[All Fields] OR "tumoral"[All Fields] OR "tumorous"[All Fields] OR "tumour"[All Fields] OR "neoplasms"[MeSH Terms] OR "neoplasms"[All Fields] OR "Tumor"[All Fields] OR "tumour s"[All Fields] OR "tumoural"[All Fields] OR "tumourous"[All Fields] OR "tumours"[All Fields] OR "tumors"[All Fields]) AND ("necrose"[All Fields] OR "necrosed"[All Fields] OR "necrosi"[All Fields] OR "necrosing"[All Fields] OR "Necrosis"[MeSH Terms] OR "Necrosis"[All Fields] OR "necroses"[All Fields]) AND ("factor a"[Supplementary Concept] OR "factor a"[All Fields])) AND ("tnf protein human"[Supplementary Concept] OR "tnf protein human"[All Fields] OR "tnf a"[All Fields])) AND "Antagonist"[Title/Abstract]) OR (((("cysts"[MeSH Terms] OR "cysts"[All Fields] OR "cyst"[All Fields] OR "neurofibroma"[MeSH Terms] OR "neurofibroma"[All Fields] OR "neurofibromas"[All Fields] OR "tumor s"[All Fields] OR "tumoral"[All Fields] OR "tumorous"[All Fields] OR "tumour"[All Fields] OR "neoplasms"[MeSH Terms] OR "neoplasms"[All Fields] OR "Tumor"[All Fields] OR "tumour s"[All Fields] OR "tumoural"[All Fields] OR "tumourous"[All Fields] OR "tumours"[All Fields] OR "tumors"[All Fields]) AND ("necrose"[All Fields] OR "necrosed"[All Fields] OR "necrosi"[All Fields] OR "necrosing"[All Fields] OR "Necrosis"[MeSH Terms] OR "Necrosis"[All Fields] OR "necroses"[All Fields]) AND ("factor a"[Supplementary Concept] OR "factor a"[All Fields])) AND ("tnf protein human"[Supplementary Concept] OR "tnf protein human"[All Fields] OR "tnf a"[All Fields])) AND "Inhibitors"[Title/Abstract]) OR (((("cysts"[MeSH Terms] OR "cysts"[All Fields] OR "cyst"[All Fields] OR "neurofibroma"[MeSH Terms] OR "neurofibroma"[All Fields] OR "neurofibromas"[All Fields] OR "tumor s"[All Fields] OR "tumoral"[All Fields] OR "tumorous"[All Fields] OR "tumour"[All Fields] OR "neoplasms"[MeSH Terms] OR "neoplasms"[All Fields] OR "Tumor"[All Fields] OR "tumour s"[All Fields] OR "tumoural"[All Fields] OR "tumourous"[All Fields] OR "tumours"[All Fields] OR "tumors"[All Fields]) AND ("necrose"[All Fields] OR "necrosed"[All Fields] OR "necrosi"[All Fields] OR "necrosing"[All Fields] OR "Necrosis"[MeSH Terms] OR "Necrosis"[All Fields] OR "necroses"[All Fields]) AND ("factor a"[Supplementary Concept] OR "factor a"[All Fields])) AND ("tnf protein human"[Supplementary Concept] OR "tnf protein human"[All Fields] OR "tnf a"[All Fields])) AND "Inhibitor"[Title/Abstract]) OR (((("cysts"[MeSH Terms] OR "cysts"[All Fields] OR "cyst"[All Fields] OR "neurofibroma"[MeSH Terms] OR "neurofibroma"[All Fields] OR "neurofibromas"[All Fields] OR "tumor s"[All Fields] OR "tumoral"[All Fields] OR "tumorous"[All Fields] OR "tumour"[All Fields] OR "neoplasms"[MeSH Terms] OR "neoplasms"[All Fields] OR "Tumor"[All Fields] OR "tumour s"[All Fields] OR "tumoural"[All Fields] OR "tumourous"[All Fields] OR "tumours"[All Fields] OR "tumors"[All Fields]) AND ("necrose"[All Fields] OR "necrosed"[All Fields] OR "necrosi"[All Fields] OR "necrosing"[All Fields] OR "Necrosis"[MeSH Terms] OR "Necrosis"[All Fields] OR "necroses"[All Fields]) AND ("factor a"[Supplementary Concept] OR "factor a"[All Fields])) AND ("tnf protein human"[Supplementary Concept] OR "tnf protein human"[All Fields] OR "tnf a"[All Fields])) AND "Blockers"[Title/Abstract]) OR (((("cysts"[MeSH Terms] OR "cysts"[All Fields] OR "cyst"[All Fields] OR "neurofibroma"[MeSH Terms] OR "neurofibroma"[All Fields] OR "neurofibromas"[All Fields] OR "tumor s"[All Fields] OR "tumoral"[All Fields] OR "tumorous"[All Fields] OR "tumour"[All Fields] OR "neoplasms"[MeSH Terms] OR "neoplasms"[All Fields] OR "Tumor"[All Fields] OR "tumour s"[All Fields] OR "tumoural"[All Fields] OR "tumourous"[All Fields] OR "tumours"[All Fields] OR "tumors"[All Fields]) AND ("necrose"[All Fields] OR "necrosed"[All Fields] OR "necrosi"[All Fields] OR "necrosing"[All Fields] OR "Necrosis"[MeSH Terms] OR "Necrosis"[All Fields] OR "necroses"[All Fields]) AND ("factor a"[Supplementary Concept] OR "factor a"[All Fields])) AND ("tnf protein human"[Supplementary Concept] OR "tnf protein human"[All Fields] OR "tnf a"[All Fields])) AND "Blocker"[Title/Abstract])) AND 1000/01/01:2025/05/30[Date - Publication])) AND 1000/01/01:2025/05/30[Date - Publication]) OR ((("Etanercept"[MeSH Terms] AND 1000/01/01:2025/05/30[Date - Publication]) OR (("tnfr fc fusion protein"[Title/Abstract] OR "fusion protein tnfr fc"[Title/Abstract] OR "tnfr fc fusion protein"[Title/Abstract] OR "TNR-001"[Title/Abstract] OR (("trinitrotoluene"[Supplementary Concept] OR "trinitrotoluene"[All Fields] OR "tnt"[All Fields] OR "trinitrotoluene"[MeSH Terms]) AND "receptor fusion protein"[Title/Abstract]) OR "TNR-001"[Title/Abstract] OR "Etanercept-szzs"[Title/Abstract] OR "tnf receptor type ii igg fusion protein"[Title/Abstract] OR "tnf receptor type ii igg fusion protein"[Title/Abstract] OR "Erelzi"[Title/Abstract] OR "recombinant human dimeric tnf receptor type ii igg fusion protein"[Title/Abstract] OR "recombinant human dimeric tnf receptor type ii igg fusion protein"[Title/Abstract] OR "Enbrel"[Title/Abstract]) AND 1000/01/01:2025/05/30[Date - Publication])) AND 1000/01/01:2025/05/30[Date - Publication]) OR ((("Adalimumab"[MeSH Terms] AND 1000/01/01:2025/05/30[Date - Publication]) OR (("Humira"[Title/Abstract] OR "Adalimumab-adbm"[Title/Abstract] OR "Amjevita"[Title/Abstract] OR "Adalimumab-atto"[Title/Abstract] OR "Cyltezo"[Title/Abstract] OR (("Adalimumab"[Supplementary Concept] OR "Adalimumab"[All Fields] OR "D2E7"[All Fields] OR "Adalimumab"[MeSH Terms]) AND "Antibody"[Title/Abstract]) OR "antibody d2e7"[Title/Abstract]) AND 1000/01/01:2025/05/30[Date - Publication])) AND 1000/01/01:2025/05/30[Date - Publication]) OR ((("Infliximab"[MeSH Terms] AND 1000/01/01:2025/05/30[Date - Publication]) OR (("mab ca2"[Title/Abstract] OR "monoclonal antibody ca2"[Title/Abstract] OR (("antibodie"[All Fields] OR "antibodies"[Supplementary Concept] OR "antibodies"[All Fields] OR "antibodies"[MeSH Terms] OR "antibody s"[All Fields] OR "antibodys"[All Fields] OR "immunoglobulins"[Supplementary Concept] OR "immunoglobulins"[All Fields] OR "Antibody"[All Fields] OR "immunoglobulins"[MeSH Terms]) AND "ca2 monoclonal"[Title/Abstract]) OR "ca2 monoclonal antibody"[Title/Abstract] OR "Infliximab-dyyb"[Title/Abstract] OR "Infliximab-dyyb"[Title/Abstract] OR "Inflectra"[Title/Abstract] OR "Remicade"[Title/Abstract] OR "Infliximab-abda"[Title/Abstract] OR "Infliximab-abda"[Title/Abstract] OR "Renflexis"[Title/Abstract]) AND 1000/01/01:2025/05/30[Date - Publication])) AND 1000/01/01:2025/05/30[Date - Publication]) OR ((("golimumab"[Supplementary Concept] AND 1000/01/01:2025/05/30[Date - Publication]) OR (("CNTO-148"[Title/Abstract] OR (("golimumab"[Supplementary Concept] OR "golimumab"[All Fields] OR "CNTO-148"[All Fields]) AND "Simponi"[Title/Abstract]) OR "Simponi"[Title/Abstract]) AND 1000/01/01:2025/05/30[Date - Publication])) AND 1000/01/01:2025/05/30[Date - Publication]) OR ((("Certolizumab Pegol"[MeSH Terms] AND 1000/01/01:2025/05/30[Date - Publication]) OR (("Cimzia"[Title/Abstract] OR "CDP870"[Title/Abstract] OR "cdp 870"[Title/Abstract]) AND 1000/01/01:2025/05/30[Date - Publication])) AND 1000/01/01:2025/05/30[Date - Publication])) AND 1000/01/01:2025/05/30[Date - Publication] AND ((("Tuberculosis"[MeSH Terms] AND 1000/01/01:2025/05/30[Date - Publication]) OR (("Tuberculoses"[Title/Abstract] OR "kochs disease"[Title/Abstract] OR "koch s disease"[Title/Abstract] OR "koch disease"[Title/Abstract] OR "mycobacterium tuberculosis infection"[Title/Abstract] OR "infection mycobacterium tuberculosis"[Title/Abstract] OR "infections mycobacterium tuberculosis"[Title/Abstract] OR "mycobacterium tuberculosis infections"[Title/Abstract]) AND 1000/01/01:2025/05/30[Date - Publication])) AND 1000/01/01:2025/05/30[Date - Publication])) AND (1000/1/1:2025/5/30[pdat]) | 729 |
|  | 22 | ("Tuberculosis"[Mesh] AND (1000/1/1:2025/05/30[pdat])) OR ((((((((Tuberculoses[Title/Abstract]) OR (Kochs Disease[Title/Abstract])) OR (Koch's Disease[Title/Abstract])) OR (Koch Disease[Title/Abstract])) OR (Mycobacterium tuberculosis Infection[Title/Abstract])) OR (Infection, Mycobacterium tuberculosis[Title/Abstract])) OR (Infections, Mycobacterium tuberculosis[Title/Abstract])) OR (Mycobacterium tuberculosis Infections[Title/Abstract]) AND (1000/1/1:2025/05/30[pdat])) Filters: from 1000/1/1 - 2025/05/30 | 214076 |
|  | 21 | (((((((Tuberculoses[Title/Abstract]) OR (Kochs Disease[Title/Abstract])) OR (Koch's Disease[Title/Abstract])) OR (Koch Disease[Title/Abstract])) OR (Mycobacterium tuberculosis Infection[Title/Abstract])) OR (Infection, Mycobacterium tuberculosis[Title/Abstract])) OR (Infections, Mycobacterium tuberculosis[Title/Abstract])) OR (Mycobacterium tuberculosis Infections[Title/Abstract]) Filters: from 1000/1/1 - 2025/05/30 | 3761 |
|  | 20 | "Tuberculosis"[Mesh] Filters: from 1000/1/1 - 2025/05/30 Sort by: Most Recent | 213249 |
|  | 19 | (((("Tumor Necrosis Factor Inhibitors"[MeSH Terms] AND 1000/01/01:2025/05/30[Date - Publication]) OR (("tumor necrosis factor blockers"[Title/Abstract] OR "tnf inhibitors"[Title/Abstract] OR "inhibitors tnf"[Title/Abstract] OR "tnf blockers"[Title/Abstract] OR "blockers tnf"[Title/Abstract] OR "tumor necrosis factor blocker"[Title/Abstract] OR "tumor necrosis factor antagonist"[Title/Abstract] OR "tumor necrosis factor inhibitor"[Title/Abstract] OR "tnf antagonist"[Title/Abstract] OR "antagonist tnf"[Title/Abstract] OR "tnf blocker"[Title/Abstract] OR (("Blocker"[All Fields] OR "blocker s"[All Fields] OR "Blockers"[All Fields]) AND "TNF"[Title/Abstract]) OR "tnf inhibitor"[Title/Abstract] OR "inhibitor tnf"[Title/Abstract] OR "tumor necrosis factor antagonists"[Title/Abstract] OR "tnf antagonists"[Title/Abstract] OR "antagonists tnf"[Title/Abstract] OR (((("cysts"[MeSH Terms] OR "cysts"[All Fields] OR "cyst"[All Fields] OR "neurofibroma"[MeSH Terms] OR "neurofibroma"[All Fields] OR "neurofibromas"[All Fields] OR "tumor s"[All Fields] OR "tumoral"[All Fields] OR "tumorous"[All Fields] OR "tumour"[All Fields] OR "neoplasms"[MeSH Terms] OR "neoplasms"[All Fields] OR "Tumor"[All Fields] OR "tumour s"[All Fields] OR "tumoural"[All Fields] OR "tumourous"[All Fields] OR "tumours"[All Fields] OR "tumors"[All Fields]) AND ("necrose"[All Fields] OR "necrosed"[All Fields] OR "necrosi"[All Fields] OR "necrosing"[All Fields] OR "Necrosis"[MeSH Terms] OR "Necrosis"[All Fields] OR "necroses"[All Fields]) AND ("factor a"[Supplementary Concept] OR "factor a"[All Fields])) AND ("tnf protein human"[Supplementary Concept] OR "tnf protein human"[All Fields] OR "tnf a"[All Fields])) AND "Antagonists"[Title/Abstract]) OR (((("cysts"[MeSH Terms] OR "cysts"[All Fields] OR "cyst"[All Fields] OR "neurofibroma"[MeSH Terms] OR "neurofibroma"[All Fields] OR "neurofibromas"[All Fields] OR "tumor s"[All Fields] OR "tumoral"[All Fields] OR "tumorous"[All Fields] OR "tumour"[All Fields] OR "neoplasms"[MeSH Terms] OR "neoplasms"[All Fields] OR "Tumor"[All Fields] OR "tumour s"[All Fields] OR "tumoural"[All Fields] OR "tumourous"[All Fields] OR "tumours"[All Fields] OR "tumors"[All Fields]) AND ("necrose"[All Fields] OR "necrosed"[All Fields] OR "necrosi"[All Fields] OR "necrosing"[All Fields] OR "Necrosis"[MeSH Terms] OR "Necrosis"[All Fields] OR "necroses"[All Fields]) AND ("factor a"[Supplementary Concept] OR "factor a"[All Fields])) AND ("tnf protein human"[Supplementary Concept] OR "tnf protein human"[All Fields] OR "tnf a"[All Fields])) AND "Antagonist"[Title/Abstract]) OR (((("cysts"[MeSH Terms] OR "cysts"[All Fields] OR "cyst"[All Fields] OR "neurofibroma"[MeSH Terms] OR "neurofibroma"[All Fields] OR "neurofibromas"[All Fields] OR "tumor s"[All Fields] OR "tumoral"[All Fields] OR "tumorous"[All Fields] OR "tumour"[All Fields] OR "neoplasms"[MeSH Terms] OR "neoplasms"[All Fields] OR "Tumor"[All Fields] OR "tumour s"[All Fields] OR "tumoural"[All Fields] OR "tumourous"[All Fields] OR "tumours"[All Fields] OR "tumors"[All Fields]) AND ("necrose"[All Fields] OR "necrosed"[All Fields] OR "necrosi"[All Fields] OR "necrosing"[All Fields] OR "Necrosis"[MeSH Terms] OR "Necrosis"[All Fields] OR "necroses"[All Fields]) AND ("factor a"[Supplementary Concept] OR "factor a"[All Fields])) AND ("tnf protein human"[Supplementary Concept] OR "tnf protein human"[All Fields] OR "tnf a"[All Fields])) AND "Inhibitors"[Title/Abstract]) OR (((("cysts"[MeSH Terms] OR "cysts"[All Fields] OR "cyst"[All Fields] OR "neurofibroma"[MeSH Terms] OR "neurofibroma"[All Fields] OR "neurofibromas"[All Fields] OR "tumor s"[All Fields] OR "tumoral"[All Fields] OR "tumorous"[All Fields] OR "tumour"[All Fields] OR "neoplasms"[MeSH Terms] OR "neoplasms"[All Fields] OR "Tumor"[All Fields] OR "tumour s"[All Fields] OR "tumoural"[All Fields] OR "tumourous"[All Fields] OR "tumours"[All Fields] OR "tumors"[All Fields]) AND ("necrose"[All Fields] OR "necrosed"[All Fields] OR "necrosi"[All Fields] OR "necrosing"[All Fields] OR "Necrosis"[MeSH Terms] OR "Necrosis"[All Fields] OR "necroses"[All Fields]) AND ("factor a"[Supplementary Concept] OR "factor a"[All Fields])) AND ("tnf protein human"[Supplementary Concept] OR "tnf protein human"[All Fields] OR "tnf a"[All Fields])) AND "Inhibitor"[Title/Abstract]) OR (((("cysts"[MeSH Terms] OR "cysts"[All Fields] OR "cyst"[All Fields] OR "neurofibroma"[MeSH Terms] OR "neurofibroma"[All Fields] OR "neurofibromas"[All Fields] OR "tumor s"[All Fields] OR "tumoral"[All Fields] OR "tumorous"[All Fields] OR "tumour"[All Fields] OR "neoplasms"[MeSH Terms] OR "neoplasms"[All Fields] OR "Tumor"[All Fields] OR "tumour s"[All Fields] OR "tumoural"[All Fields] OR "tumourous"[All Fields] OR "tumours"[All Fields] OR "tumors"[All Fields]) AND ("necrose"[All Fields] OR "necrosed"[All Fields] OR "necrosi"[All Fields] OR "necrosing"[All Fields] OR "Necrosis"[MeSH Terms] OR "Necrosis"[All Fields] OR "necroses"[All Fields]) AND ("factor a"[Supplementary Concept] OR "factor a"[All Fields])) AND ("tnf protein human"[Supplementary Concept] OR "tnf protein human"[All Fields] OR "tnf a"[All Fields])) AND "Blockers"[Title/Abstract]) OR (((("cysts"[MeSH Terms] OR "cysts"[All Fields] OR "cyst"[All Fields] OR "neurofibroma"[MeSH Terms] OR "neurofibroma"[All Fields] OR "neurofibromas"[All Fields] OR "tumor s"[All Fields] OR "tumoral"[All Fields] OR "tumorous"[All Fields] OR "tumour"[All Fields] OR "neoplasms"[MeSH Terms] OR "neoplasms"[All Fields] OR "Tumor"[All Fields] OR "tumour s"[All Fields] OR "tumoural"[All Fields] OR "tumourous"[All Fields] OR "tumours"[All Fields] OR "tumors"[All Fields]) AND ("necrose"[All Fields] OR "necrosed"[All Fields] OR "necrosi"[All Fields] OR "necrosing"[All Fields] OR "Necrosis"[MeSH Terms] OR "Necrosis"[All Fields] OR "necroses"[All Fields]) AND ("factor a"[Supplementary Concept] OR "factor a"[All Fields])) AND ("tnf protein human"[Supplementary Concept] OR "tnf protein human"[All Fields] OR "tnf a"[All Fields])) AND "Blocker"[Title/Abstract])) AND 1000/01/01:2025/05/30[Date - Publication])) AND 1000/01/01:2025/05/30[Date - Publication]) OR ((("Etanercept"[MeSH Terms] AND 1000/01/01:2025/05/30[Date - Publication]) OR (("tnfr fc fusion protein"[Title/Abstract] OR "fusion protein tnfr fc"[Title/Abstract] OR "tnfr fc fusion protein"[Title/Abstract] OR "TNR-001"[Title/Abstract] OR (("trinitrotoluene"[Supplementary Concept] OR "trinitrotoluene"[All Fields] OR "tnt"[All Fields] OR "trinitrotoluene"[MeSH Terms]) AND "receptor fusion protein"[Title/Abstract]) OR "TNR-001"[Title/Abstract] OR "Etanercept-szzs"[Title/Abstract] OR "tnf receptor type ii igg fusion protein"[Title/Abstract] OR "tnf receptor type ii igg fusion protein"[Title/Abstract] OR "Erelzi"[Title/Abstract] OR "recombinant human dimeric tnf receptor type ii igg fusion protein"[Title/Abstract] OR "recombinant human dimeric tnf receptor type ii igg fusion protein"[Title/Abstract] OR "Enbrel"[Title/Abstract]) AND 1000/01/01:2025/05/30[Date - Publication])) AND 1000/01/01:2025/05/30[Date - Publication]) OR ((("Adalimumab"[MeSH Terms] AND 1000/01/01:2025/05/30[Date - Publication]) OR (("Humira"[Title/Abstract] OR "Adalimumab-adbm"[Title/Abstract] OR "Amjevita"[Title/Abstract] OR "Adalimumab-atto"[Title/Abstract] OR "Cyltezo"[Title/Abstract] OR (("Adalimumab"[Supplementary Concept] OR "Adalimumab"[All Fields] OR "D2E7"[All Fields] OR "Adalimumab"[MeSH Terms]) AND "Antibody"[Title/Abstract]) OR "antibody d2e7"[Title/Abstract]) AND 1000/01/01:2025/05/30[Date - Publication])) AND 1000/01/01:2025/05/30[Date - Publication]) OR ((("Infliximab"[MeSH Terms] AND 1000/01/01:2025/05/30[Date - Publication]) OR (("mab ca2"[Title/Abstract] OR "monoclonal antibody ca2"[Title/Abstract] OR (("antibodie"[All Fields] OR "antibodies"[Supplementary Concept] OR "antibodies"[All Fields] OR "antibodies"[MeSH Terms] OR "antibody s"[All Fields] OR "antibodys"[All Fields] OR "immunoglobulins"[Supplementary Concept] OR "immunoglobulins"[All Fields] OR "Antibody"[All Fields] OR "immunoglobulins"[MeSH Terms]) AND "ca2 monoclonal"[Title/Abstract]) OR "ca2 monoclonal antibody"[Title/Abstract] OR "Infliximab-dyyb"[Title/Abstract] OR "Infliximab-dyyb"[Title/Abstract] OR "Inflectra"[Title/Abstract] OR "Remicade"[Title/Abstract] OR "Infliximab-abda"[Title/Abstract] OR "Infliximab-abda"[Title/Abstract] OR "Renflexis"[Title/Abstract]) AND 1000/01/01:2025/05/30[Date - Publication])) AND 1000/01/01:2025/05/30[Date - Publication]) OR ((("golimumab"[Supplementary Concept] AND 1000/01/01:2025/05/30[Date - Publication]) OR (("CNTO-148"[Title/Abstract] OR (("golimumab"[Supplementary Concept] OR "golimumab"[All Fields] OR "CNTO-148"[All Fields]) AND "Simponi"[Title/Abstract]) OR "Simponi"[Title/Abstract]) AND 1000/01/01:2025/05/30[Date - Publication])) AND 1000/01/01:2025/05/30[Date - Publication]) OR ((("Certolizumab Pegol"[MeSH Terms] AND 1000/01/01:2025/05/30[Date - Publication]) OR (("Cimzia"[Title/Abstract] OR "CDP870"[Title/Abstract] OR "cdp 870"[Title/Abstract]) AND 1000/01/01:2025/05/30[Date - Publication])) AND 1000/01/01:2025/05/30[Date - Publication])) AND (1000/1/1:2025/5/30[pdat]) | 30112 |
|  | 18 | ("Certolizumab Pegol"[Mesh] AND (1000/1/1:2025/05/30[pdat])) OR (((Cimzia[Title/Abstract]) OR (CDP870[Title/Abstract])) OR (CDP 870[Title/Abstract]) AND (1000/1/1:2025/05/30[pdat])) Filters: from 1000/1/1 - 2025/05/30 | 844 |
|  | 17 | ((Cimzia[Title/Abstract]) OR (CDP870[Title/Abstract])) OR (CDP 870[Title/Abstract]) Filters: from 1000/1/1 - 2025/05/30 | 103 |
|  | 16 | "Certolizumab Pegol"[Mesh] Filters: from 1000/1/1 - 2025/05/30 Sort by: Most Recent | 784 |
|  | 15 | ("golimumab" [Supplementary Concept] AND (1000/1/1:2025/05/30[pdat])) OR (((CNTO-148[Title/Abstract]) OR (CNTO 148 Simponi[Title/Abstract])) OR (Simponi[Title/Abstract]) AND (1000/1/1:2025/05/30[pdat])) Filters: from 1000/1/1 - 2025/05/30 | 874 |
|  | 14 | ((CNTO-148[Title/Abstract]) OR (CNTO 148 Simponi[Title/Abstract])) OR (Simponi[Title/Abstract]) Filters: from 1000/1/1 - 2025/05/30 | 51 |
|  | 13 | "golimumab" [Supplementary Concept] Filters: from 1000/1/1 - 2025/05/30 Sort by: Most Recent | 844 |
|  | 12 | ("Infliximab"[Mesh] AND (1000/1/1:2025/05/30[pdat])) OR (((((((((((MAb cA2[Title/Abstract]) OR (Monoclonal Antibody cA2[Title/Abstract])) OR (Antibody cA2, Monoclonal[Title/Abstract])) OR (cA2, Monoclonal Antibody[Title/Abstract])) OR (Infliximab-dyyb[Title/Abstract])) OR (Infliximab dyyb[Title/Abstract])) OR (Inflectra[Title/Abstract])) OR (Remicade[Title/Abstract])) OR (Infliximab-abda[Title/Abstract])) OR (Infliximab abda[Title/Abstract])) OR (Renflexis[Title/Abstract]) AND (1000/1/1:2025/05/30[pdat])) Filters: from 1000/1/1 - 2025/05/30 | 12921 |
|  | 11 | ((((((((((MAb cA2[Title/Abstract]) OR (Monoclonal Antibody cA2[Title/Abstract])) OR (Antibody cA2, Monoclonal[Title/Abstract])) OR (cA2, Monoclonal Antibody[Title/Abstract])) OR (Infliximab-dyyb[Title/Abstract])) OR (Infliximab dyyb[Title/Abstract])) OR (Inflectra[Title/Abstract])) OR (Remicade[Title/Abstract])) OR (Infliximab-abda[Title/Abstract])) OR (Infliximab abda[Title/Abstract])) OR (Renflexis[Title/Abstract]) Filters: from 1000/1/1 - 2025/05/30 | 565 |
|  | 10 | "Infliximab"[Mesh] Filters: from 1000/1/1 - 2025/05/30 Sort by: Most Recent | 12718 |
|  | 9 | ("Adalimumab"[Mesh] AND (1000/1/1:2025/05/30[pdat])) OR (((((((Humira[Title/Abstract]) OR (Adalimumab-adbm[Title/Abstract])) OR (Amjevita[Title/Abstract])) OR (Adalimumab-atto[Title/Abstract])) OR (Cyltezo[Title/Abstract])) OR (D2E7 Antibody[Title/Abstract])) OR (Antibody, D2E7[Title/Abstract]) AND (1000/1/1:2025/05/30[pdat])) Filters: from 1000/1/1 - 2025/05/30 | 8333 |
|  | 8 | ((((((Humira[Title/Abstract]) OR (Adalimumab-adbm[Title/Abstract])) OR (Amjevita[Title/Abstract])) OR (Adalimumab-atto[Title/Abstract])) OR (Cyltezo[Title/Abstract])) OR (D2E7 Antibody[Title/Abstract])) OR (Antibody, D2E7[Title/Abstract]) Filters: from 1000/1/1 - 2025/05/30 | 1881 |
|  | 7 | "Adalimumab"[Mesh] Filters: from 1000/1/1 - 2025/05/30 | 7586 |
|  | 6 | ("Etanercept"[Mesh] AND (1000/1/1:2025/05/30[pdat])) OR (((((((((((((((TNFR-Fc Fusion Protein[Title/Abstract]) OR (Fusion Protein, TNFR-Fc[Title/Abstract])) OR (TNFR Fc Fusion Protein[Title/Abstract])) OR (TNR 001[Title/Abstract])) OR (TNT Receptor Fusion Protein[Title/Abstract])) OR (TNTR-Fc[Title/Abstract])) OR (TNR-001[Title/Abstract])) OR (TNR001[Title/Abstract])) OR (Etanercept-szzs[Title/Abstract])) OR (TNF Receptor Type II-IgG Fusion Protein[Title/Abstract])) OR (TNF Receptor Type II IgG Fusion Protein[Title/Abstract])) OR (Erelzi[Title/Abstract])) OR (Recombinant Human Dimeric TNF Receptor Type II-IgG Fusion Protein[Title/Abstract])) OR (Recombinant Human Dimeric TNF Receptor Type II IgG Fusion Protein[Title/Abstract])) OR (Enbrel[Title/Abstract]) AND (1000/1/1:2025/05/30[pdat])) Filters: from 1000/1/1 - 2025/05/30 | 6881 |
|  | 5 | ((((((((((((((TNFR-Fc Fusion Protein[Title/Abstract]) OR (Fusion Protein, TNFR-Fc[Title/Abstract])) OR (TNFR Fc Fusion Protein[Title/Abstract])) OR (TNR 001[Title/Abstract])) OR (TNT Receptor Fusion Protein[Title/Abstract])) OR (TNTR-Fc[Title/Abstract])) OR (TNR-001[Title/Abstract])) OR (TNR001[Title/Abstract])) OR (Etanercept-szzs[Title/Abstract])) OR (TNF Receptor Type II-IgG Fusion Protein[Title/Abstract])) OR (TNF Receptor Type II IgG Fusion Protein[Title/Abstract])) OR (Erelzi[Title/Abstract])) OR (Recombinant Human Dimeric TNF Receptor Type II-IgG Fusion Protein[Title/Abstract])) OR (Recombinant Human Dimeric TNF Receptor Type II IgG Fusion Protein[Title/Abstract])) OR (Enbrel[Title/Abstract]) Filters: from 1000/1/1 - 2025/05/30 | 415 |
|  | 4 | "Etanercept"[Mesh] Filters: from 1000/1/1 - 2025/05/30 | 6700 |
|  | 3 | ("Tumor Necrosis Factor Inhibitors"[Mesh] AND (1000/1/1:2025/05/30[pdat])) OR (((((((((((((((((((((((Tumor Necrosis Factor Blockers[Title/Abstract]) OR (TNF Inhibitors[Title/Abstract])) OR (Inhibitors, TNF[Title/Abstract])) OR (TNF Blockers[Title/Abstract])) OR (Blockers, TNF[Title/Abstract])) OR (Tumor Necrosis Factor Blocker[Title/Abstract])) OR (Tumor Necrosis Factor Antagonist[Title/Abstract])) OR (Tumor Necrosis Factor Inhibitor[Title/Abstract])) OR (TNF Antagonist[Title/Abstract])) OR (Antagonist, TNF[Title/Abstract])) OR (TNF Blocker[Title/Abstract])) OR (Blocker, TNF[Title/Abstract])) OR (TNF Inhibitor[Title/Abstract])) OR (Inhibitor, TNF[Title/Abstract])) OR (Tumor Necrosis Factor Antagonists[Title/Abstract])) OR (TNF Antagonists[Title/Abstract])) OR (Antagonists, TNF[Title/Abstract])) OR (Tumor Necrosis Factor-a (TNF-a) Antagonists[Title/Abstract])) OR (Tumor Necrosis Factor-a (TNF-a) Antagonist[Title/Abstract])) OR (Tumor Necrosis Factor-a (TNF-a) Inhibitors[Title/Abstract])) OR (Tumor Necrosis Factor-a (TNF-a) Inhibitor[Title/Abstract])) OR (Tumor Necrosis Factor-a (TNF-a) Blockers[Title/Abstract])) OR (Tumor Necrosis Factor-a (TNF-a) Blocker[Title/Abstract]) AND (1000/1/1:2025/05/30[pdat])) Filters: from 1000/1/1 - 2025/05/30 | 9777 |
|  | 2 | ((((((((((((((((((((((Tumor Necrosis Factor Blockers[Title/Abstract]) OR (TNF Inhibitors[Title/Abstract])) OR (Inhibitors, TNF[Title/Abstract])) OR (TNF Blockers[Title/Abstract])) OR (Blockers, TNF[Title/Abstract])) OR (Tumor Necrosis Factor Blocker[Title/Abstract])) OR (Tumor Necrosis Factor Antagonist[Title/Abstract])) OR (Tumor Necrosis Factor Inhibitor[Title/Abstract])) OR (TNF Antagonist[Title/Abstract])) OR (Antagonist, TNF[Title/Abstract])) OR (TNF Blocker[Title/Abstract])) OR (Blocker, TNF[Title/Abstract])) OR (TNF Inhibitor[Title/Abstract])) OR (Inhibitor, TNF[Title/Abstract])) OR (Tumor Necrosis Factor Antagonists[Title/Abstract])) OR (TNF Antagonists[Title/Abstract])) OR (Antagonists, TNF[Title/Abstract])) OR (Tumor Necrosis Factor-a (TNF-a) Antagonists[Title/Abstract])) OR (Tumor Necrosis Factor-a (TNF-a) Antagonist[Title/Abstract])) OR (Tumor Necrosis Factor-a (TNF-a) Inhibitors[Title/Abstract])) OR (Tumor Necrosis Factor-a (TNF-a) Inhibitor[Title/Abstract])) OR (Tumor Necrosis Factor-a (TNF-a) Blockers[Title/Abstract])) OR (Tumor Necrosis Factor-a (TNF-a) Blocker[Title/Abstract]) Filters: from 1000/1/1 - 2025/05/30 | 7543 |
|  | 1 | "Tumor Necrosis Factor Inhibitors"[Mesh] Filters: from 1000/1/1 - 2025/05/30 | 3093 |
| **Web of Science** | #9 | #8 AND #7 | 4319 |
|  | #8 | TS=(tuberculosis or Tuberculoses or Kochs Disease or Koch's Disease or Koch Disease or Mycobacterium tuberculosis Infection or Infection, Mycobacterium tuberculosis or Infections, Mycobacterium tuberculosis or Mycobacterium tuberculosis Infections) | 438133 |
|  | #7 | #6 OR #5 OR #4 OR #3 OR #2 OR #1 | 217756 |
|  | #6 | TS=(certolizumab pegol or Cimzia or CDP870 or CDP 870) | 3514 |
|  | #5 | TS=(golimumab or CNTO-148 or CNTO 148 or Simponi) | 4264 |
|  | #4 | TS=(infliximab or MAb cA2 or Monoclonal Antibody cA2 or Antibody cA2, Monoclonal or cA2, Monoclonal Antibody or Infliximab-dyyb or Infliximab dyyb or Inflectra or Remicade or Infliximab-abda or Infliximab abda or Renflexis) | 48262 |
|  | #3 | TS=(adalimumab or Humira or Adalimumab-adbm or Amjevita or Adalimumab-atto or Cyltezo or D2E7 Antibody or Antibody, D2E7) | 27058 |
|  | #2 | TS=(etanercept or TNFR-Fc Fusion Protein or Fusion Protein, TNFR-Fc or TNFR Fc Fusion Protein or TNR 001 or TNT Receptor Fusion Protein or TNTR-Fc or TNR-001 or TNR001 or Etanercept-szzs or TNF Receptor Type II-IgG Fusion Protein or TNF Receptor Type II IgG Fusion Protein or Erelzi or Recombinant Human Dimeric TNF Receptor Type II-IgG Fusion Protein or Recombinant Human Dimeric TNF Receptor Type II IgG Fusion Protein or Enbrel) | 22526 |
|  | #1 | TS=(Tumor Necrosis Factor Inhibitors or Tumor Necrosis Factor Blockers or TNF Inhibitors or Inhibitors, TNF or TNF Blockers or Blockers, TNF or Tumor Necrosis Factor Blocker or Tumor Necrosis Factor Antagonist or Tumor Necrosis Factor Inhibitor or TNF Antagonist or Antagonist, TNF or TNF Blocker or Blocker, TNF or TNF Inhibitor or Inhibitor, TNF or Tumor Necrosis Factor Antagonists or TNF Antagonists or Antagonists, TNF or Tumor Necrosis Factor-a (TNF-a) Antagonists or Tumor Necrosis Factor-a (TNF-a) Antagonist or Tumor Necrosis Factor-a (TNF-a) Inhibitors or Tumor Necrosis Factor-a (TNF-a) Inhibitor or Tumor Necrosis Factor-a (TNF-a) Blockers or Tumor Necrosis Factor-a (TNF-a) Blocker) | 161934 |
| **Embase** | #23 | #19 AND #22 | 6928 |
|  | #22 | #20 OR #21 | 320666 |
|  | #21 | 'tuberculoses':ab,ti OR 'kochs disease':ab,ti OR 'koch disease':ab,ti OR 'mycobacterium tuberculosis infection':ab,ti OR 'infection, mycobacterium tuberculosis':ab,ti OR 'infections, mycobacterium tuberculosis':ab,ti OR 'mycobacterium tuberculosis infections':ab,ti | 4522 |
|  | 20 | 'tuberculosis'/exp | 320128 |
|  | #19 | #3 OR #6 OR #9 OR #12 OR #15 OR #18 | 143306 |
|  | #18 | #16 OR #17 | 10481 |
|  | #17 | ‘Cimzia’:ab,ti or ‘CDP870’:ab,ti or ‘CDP 870’:ab,ti | 154 |
|  | #16 | 'certolizumab pegol'/exp | 10468 |
|  | #15 | #13 OR #14 | 11709 |
|  | #14 | ‘CNTO-148’:ab,ti or ‘CNTO 148’:ab,ti or ‘Simponi’:ab,ti | 79 |
|  | #13 | 'golimumab'/exp | 11707 |
|  | #12 | #10 OR #11 | 69645 |
|  | #11 | ‘MAb cA2’:ab,ti or ‘Monoclonal Antibody cA2’:ab,ti or ‘Antibody cA2, Monoclonal’:ab,ti or ‘cA2, Monoclonal Antibody’:ab,ti | 29 |
|  | #10 | 'infliximab'/exp | 69621 |
|  | #9 | #7 OR #8 | 52050 |
|  | #8 | 'humira':ab,ti OR 'adalimumab-adbm':ab,ti OR 'amjevita':ab,ti OR 'adalimumab-atto':ab,ti OR 'cyltezo':ab,ti | 981 |
|  | #7 | 'adalimumab'/exp | 52002 |
|  | #6 | #4 OR #5 | 40696 |
|  | #5 | 'tnfr-fc fusion protein':ab,ti OR 'fusion protein, tnfr-fc':ab,ti OR 'tnfr fc fusion protein':ab,ti OR 'tnr 001':ab,ti OR 'tnt receptor fusion protein':ab,ti OR 'tntr-fc':ab,ti OR 'tnr-001':ab,ti OR 'tnr001':ab,ti OR 'etanercept-szzs':ab,ti OR 'tnf receptor type ii-igg fusion protein':ab,ti OR 'tnf receptor type ii igg fusion protein':ab,ti OR 'erelzi':ab,ti OR 'recombinant human dimeric tnf receptor type ii-igg fusion protein':ab,ti OR 'recombinant human dimeric tnf receptor type ii igg fusion protein':ab,ti OR 'enbrel':ab,ti | 777 |
|  | #4 | 'etanercept'/exp | 40592 |
|  | #3 | #1 OR #2 | 143159 |
|  | #2 | 'tumor necrosis factor blockers':ab,ti OR 'tnf inhibitors':ab,ti OR 'inhibitors, tnf':ab,ti OR 'tnf blockers':ab,ti OR 'blockers, tnf':ab,ti OR 'tumor necrosis factor blocker':ab,ti OR 'tumor necrosis factor antagonist':ab,ti OR 'tumor necrosis factor inhibitor':ab,ti OR 'tnf antagonist':ab,ti OR 'antagonist, tnf':ab,ti OR 'tnf blocker':ab,ti OR 'tnf inhibitor':ab,ti OR 'blocker, tnf':ab,ti OR 'inhibitor, tnf':ab,ti OR 'tumor necrosis factor antagonists':ab,ti OR 'tnf antagonists':ab,ti OR 'antagonists, tnf':ab,ti OR 'tumor necrosis factor- a (tnf-a) antagonists':ab,ti OR 'tumor necrosis factor- a (tnf-a) antagonist':ab,ti OR 'tumor necrosis factor- a (tnf- a) inhibitors':ab,ti OR 'tumor necrosis factor-a (tnf- a) inhibitor':ab,ti OR 'tumor necrosis factor-a (tnf- a) blockers':ab,ti | 11188 |
|  | #1 | 'tumor necrosis factor inhibitor'/exp | 141369 |
| **Cochrane Library** | #21 | #17 AND #20 | 33 |
|  | #20 | #18 OR #19 | 3761 |
|  | #19 | (Tuberculoses):ti,ab,kw or(Kochs Disease):ti,ab,kw or(Koch's Disease):ti,ab,kw or(Koch Disease):ti,ab,kw or(Mycobacterium tuberculosis Infection):ti,ab,kw or(Infection, Mycobacterium tuberculosis):ti,ab,kw or(Infections, Mycobacterium tuberculosis):ti,ab,kw or(Mycobacterium tuberculosis Infections):ti,ab,kw | 671 |
|  | #18 | MeSH descriptor: [tuberculosis] explode all trees | 3480 |
|  | #17 | #3 OR #6 OR #9 OR #12 OR #13 OR #16 | 8552 |
|  | #16 | #14 OR #15 | 270 |
|  | #15 | (Cimzia):ti,ab,kw or(CDP870):ti,ab,kw or(CDP 870):ti,ab,kw | 80 |
|  | #14 | MeSH descriptor: [certolizumab pegol] explode all trees | 217 |
|  | #13 | (golimumab):ti,ab,kw or(CNTO-148):ti,ab,kw or(CNTO 148):ti,ab,kw or(Simponi):ti,ab,kw | 825 |
|  | #12 | #10 OR #11 | 1264 |
|  | #11 | (MAb cA2):ti,ab,kw or(Monoclonal Antibody cA2):ti,ab,kw or(Antibody cA2, Monoclonal):ti,ab,kw or(cA2, Monoclonal Antibody):ti,ab,kw or(Infliximab-dyyb):ti,ab,kw or(Infliximab dyyb):ti,ab,kw or(Inflectra):ti,ab,kw or(Remicade):ti,ab,kw or(Infliximab-abda):ti,ab,kw or(Infliximab abda):ti,ab,kw or(Renflexis):ti,ab,kw | 288 |
|  | #10 | MeSH descriptor: [infliximab] explode all trees | 1081 |
|  | #9 | #7 OR #8 | 1502 |
|  | #8 | (Humira):ti,ab,kw or(Adalimumab-adbm):ti,ab,kw or(Amjevita):ti,ab,kw or(Adalimumab-atto):ti,ab,kw or(Cyltezo):ti,ab,kw or(D2E7 Antibody):ti,ab,kw or(Antibody, D2E7):ti,ab,kw | 470 |
|  | #7 | MeSH descriptor: [adalimumab] explode all trees | 1153 |
|  | #6 | #4 OR #5 | 1203 |
|  | #5 | (TNFR-Fc Fusion Protein):ti,ab,kw or (Fusion Protein, TNFR-Fc):ti,ab,kw or (TNFR Fc Fusion Protein):ti,ab,kw or(TNR 001):ti,ab,kw or(TNT Receptor Fusion Protein):ti,ab,kw or(TNTR-Fc):ti,ab,kw or(TNR-001):ti,ab,kw or(TNR001):ti,ab,kw or(Etanercept-szzs):ti,ab,kw or(TNF Receptor Type II-IgG Fusion Protein):ti,ab,kw or(TNF Receptor Type II IgG Fusion Protein):ti,ab,kw or(Erelzi):ti,ab,kw or(Recombinant Human Dimeric TNF Receptor Type II-IgG Fusion Protein):ti,ab,kw or(Recombinant Human Dimeric TNF Receptor Type II IgG Fusion Protein):ti,ab,kw or(Enbrel):ti,ab,kw | 304 |
|  | #4 | MeSH descriptor: [etanercept] explode all trees | 1001 |
|  | #3 | #1 OR #2 | 4990 |
|  | #2 | (Tumor Necrosis Factor Blockers):ti,ab,kw or (TNF Inhibitors):ti,ab,kw or (Inhibitors, TNF):ti,ab,kw or (TNF Blockers):ti,ab,kw or (Blockers, TNF):ti,ab,kw or (Tumor Necrosis Factor Blocker):ti,ab,kw or (Tumor Necrosis Factor Antagonist):ti,ab,kw or (Tumor Necrosis Factor Inhibitor):ti,ab,kw or (TNF Antagonist):ti,ab,kw or (Antagonist, TNF):ti,ab,kw or (TNF Blocker):ti,ab,kw or (TNF Inhibitor):ti,ab,kw or (Blocker, TNF):ti,ab,kw or (Inhibitor, TNF):ti,ab,kw or (Tumor Necrosis Factor Antagonists):ti,ab,kw or (TNF Antagonists):ti,ab,kw or (Antagonists, TNF):ti,ab,kw or (Tumor Necrosis Factor-a (TNF-a) Antagonists):ti,ab,kw or (Tumor Necrosis Factor-a (TNF-a) Antagonist):ti,ab,kw or (Tumor Necrosis Factor-a (TNF-a) Inhibitors):ti,ab,kw or (Tumor Necrosis Factor-a (TNF-a) Inhibitor):ti,ab,kw or (Tumor Necrosis Factor-a (TNF-a) Blockers):ti,ab,kw | 4941 |
|  | #1 | MeSH descriptor: [Tumor Necrosis Factor Inhibitor] explode all trees | 173 |


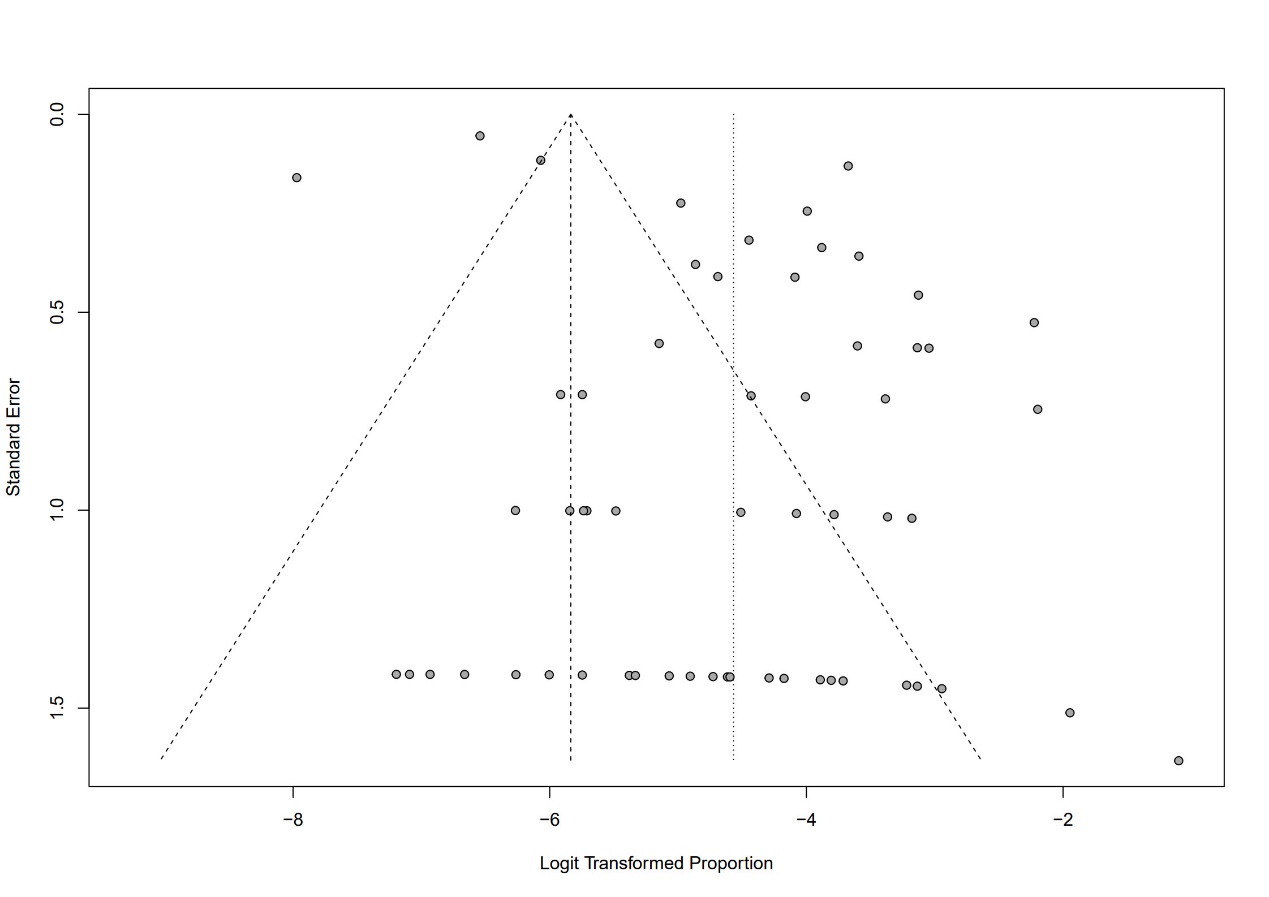


Figure S1. Funnel plots of the effects of intervention arms on tuberculosis (TB). (Linear regression test of funnel plot asymmetry. *P* = 0.0002)


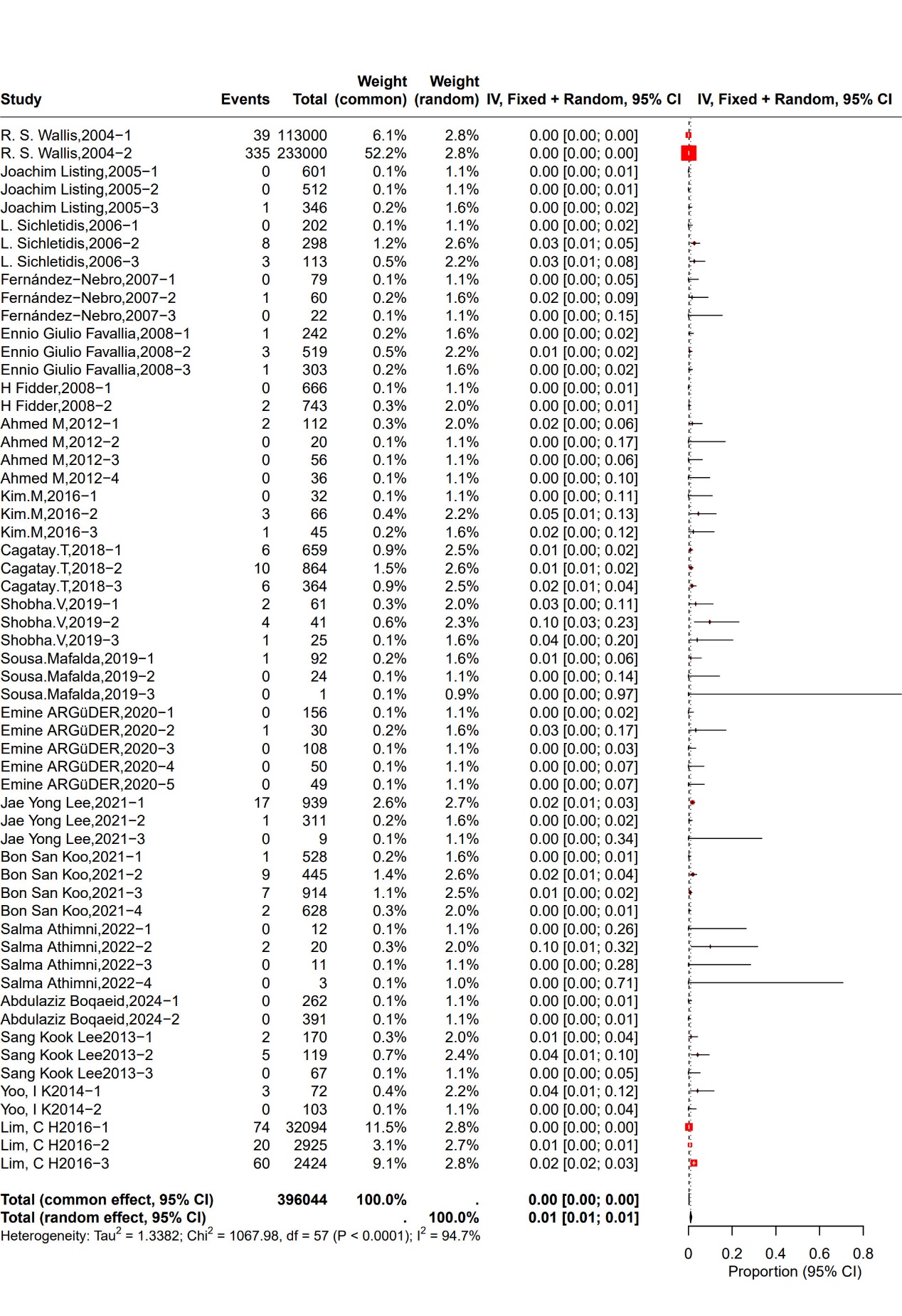
Figure S2. Forest plots from pairwise meta-analyses of the relative risk (RR) for tuberculosis (TB) treatment outcomes.

.


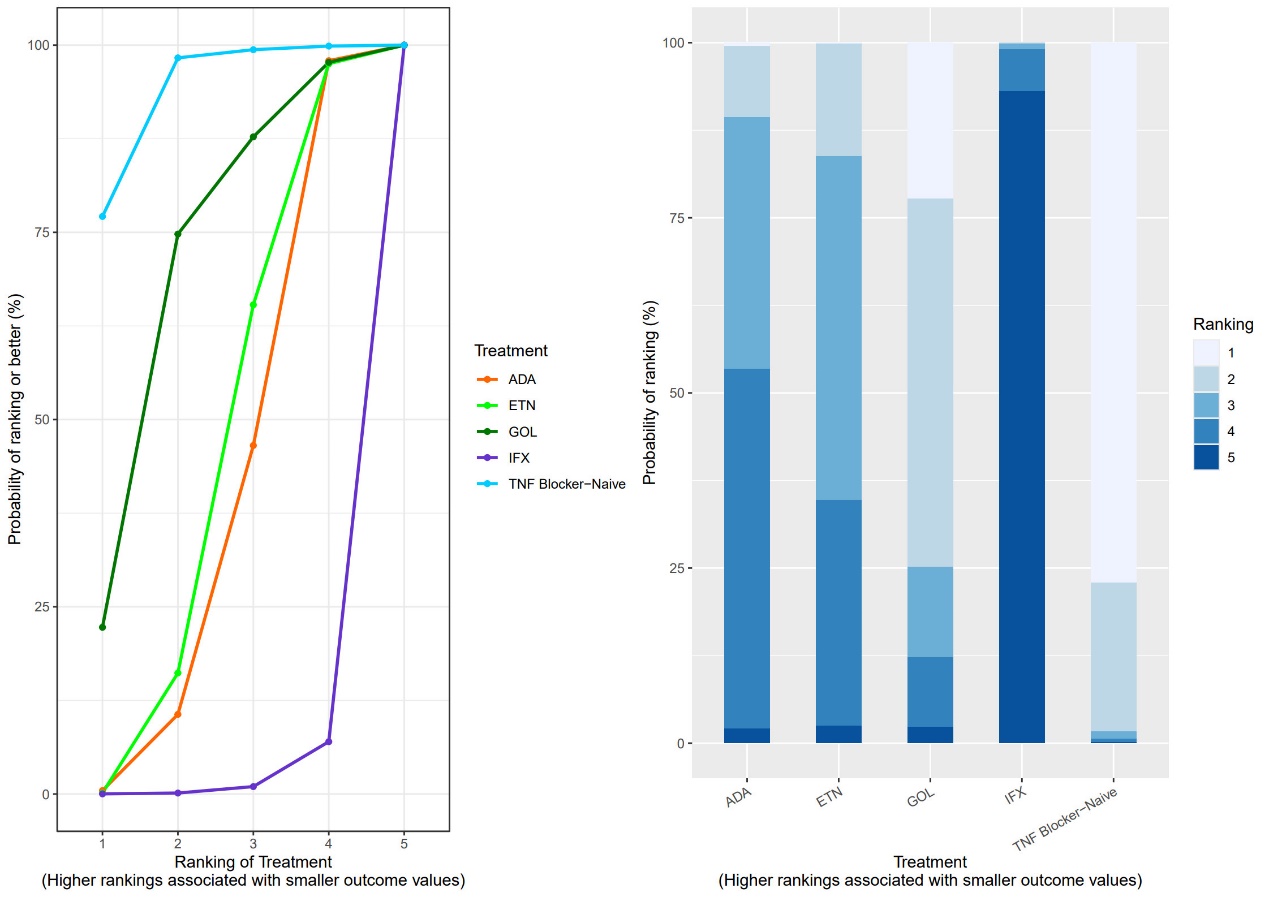


Figure S3. Surface under the cumulative ranking curve (SUCRA) and treatment rank probabilities for tuberculosis (TB) among patients with a follow-up duration of less than 2 years. Treatments: ETN: Etanercept, ADA: Adalimumab, IFX: Infliximab, GOL: Golimumab, CZP: Certolizumab pegol; TB: Tuberculosis; SUCRA: surface under the cumulative ranking curve


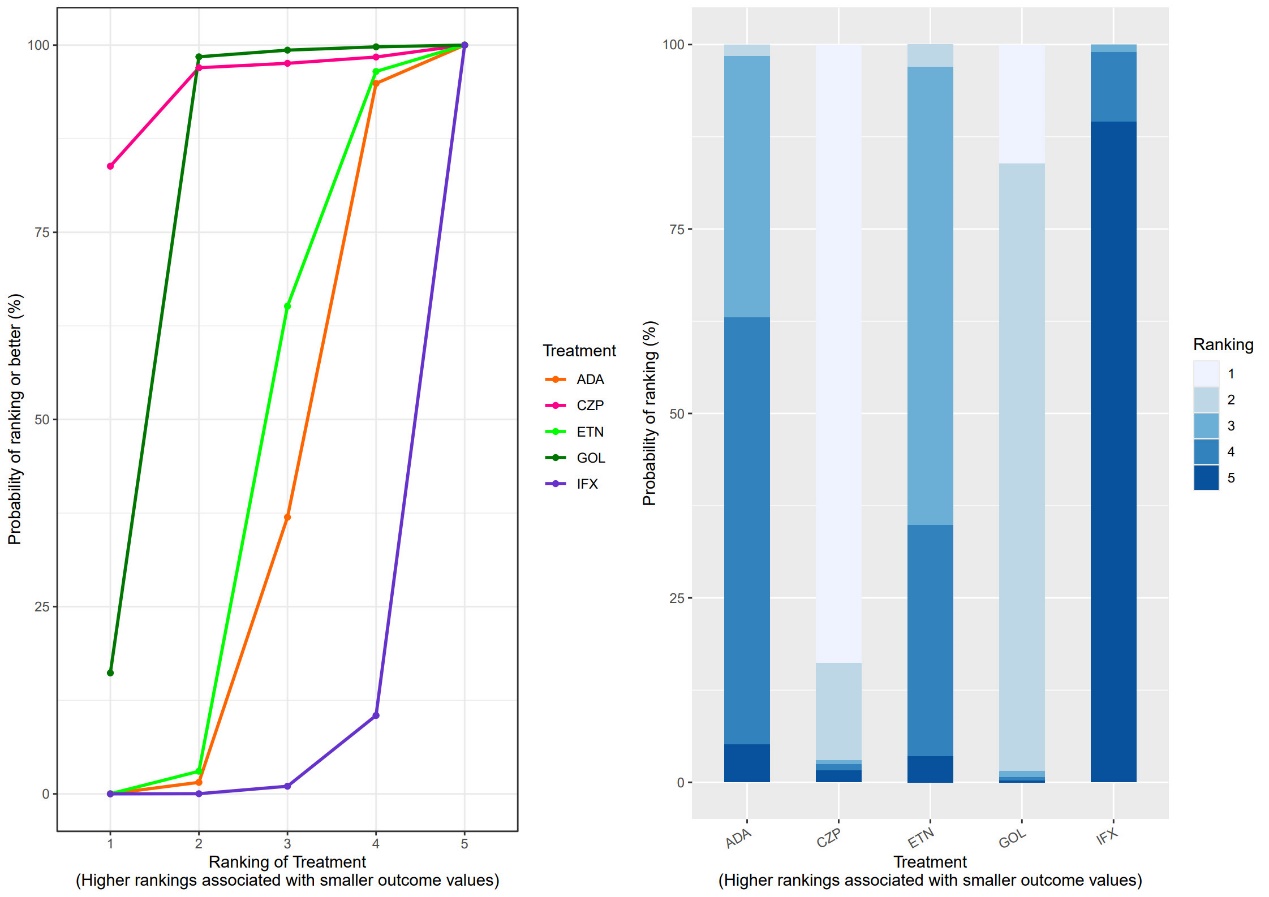


Figure S4. Surface under the cumulative ranking curve (SUCRA) and treatment rank probabilities for tuberculosis (TB) among patients with prophylactic anti-TB therapy. Treatments: ETN: Etanercept, ADA: Adalimumab, IFX: Infliximab, GOL: Golimumab, CZP: Certolizumab pegol; TB: Tuberculosis; SUCRA: surface under the cumulative ranking curve


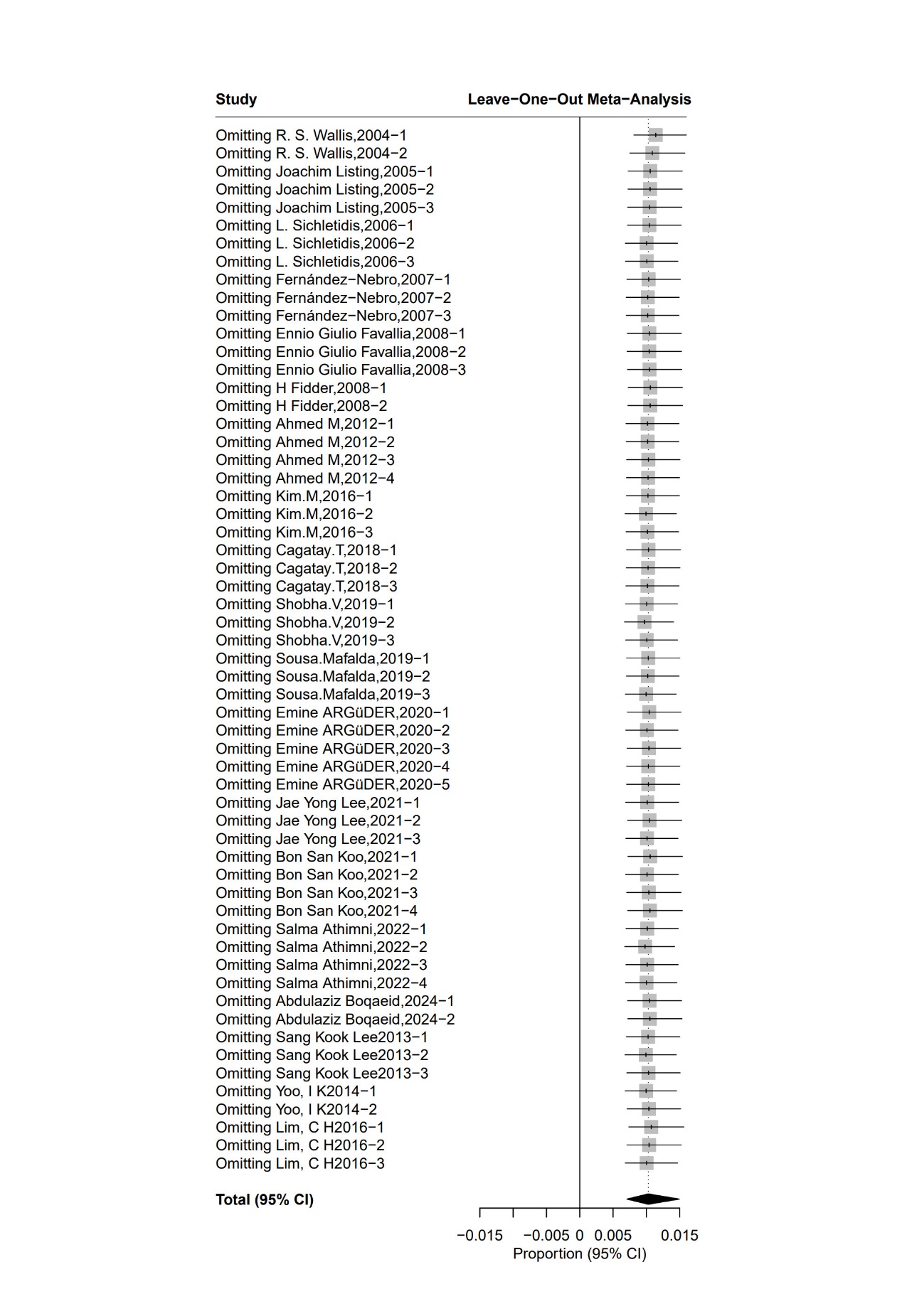


Figure S5. Sensitivity analysis of the meta-analysis results for tuberculosis (TB) treatment: sequential omission of individual intervention pairs.
